# Optimising self-care support for people with heart failure and their caregivers: development of the Rehabilitation Enablement in Chronic Heart Failure (REACH-HF) intervention using intervention mapping

**DOI:** 10.1186/s40814-016-0075-x

**Published:** 2016-08-02

**Authors:** Colin J. Greaves, Jennifer Wingham, Carolyn Deighan, Patrick Doherty, Jennifer Elliott, Wendy Armitage, Michelle Clark, Jackie Austin, Charles Abraham, Julia Frost, Sally Singh, Kate Jolly, Kevin Paul, Louise Taylor, Sarah Buckingham, Russell Davis, Hasnain Dalal, Rod S. Taylor, on behalf of the REACH-HF investigators, Rod S. Taylor, Hasnain Dalal, Charles Abraham, Jackie Austin, Nicky Britten, Sarah Buckingham, Russell Davis, Patrick Doherty, Lorna Geach, Colin J. Greaves, Colin Green, Kate Jolly, Kevin Paul, Chris Hayward, Victoria Eyre, Sally Singh, Robin van Lingen, Jennifer Wingham

**Affiliations:** 1 Institute for Health Research, University of Exeter Medical School, St Luke’s Campus, Magdalen Road, Exeter, EX1 2LU UK; 2 Research, Development & Innovation, Royal Cornwall Hospitals NHS Trust, Knowledge Spa, Royal Cornwall Hospital, Truro, Cornwall TR1 3HD UK; 3 Heart Manual Department, NHS Lothian Heart Manual Department, Astley Ainslie Hospital, 133 Grange Loan, Edinburgh, EH9 2HL UK; 4 Department of Health Sciences, University of York, Area 4, Seebohm Rowntree Building, York, YO10 5DD UK; 5 Chest Heart & Stroke Scotland, 3rd floor, Rosebery House, 9 Haymarket Terrace, Edinburgh, EH12 5EZ UK; 6 Heart Failure and Cardiac Rehabilitation Services, Aneurin Bevan Health Board, Ty-Meddyg, Nevill Hall Hospital, Abergavenny, Gwent NP7 7EG UK; 7 University Hospitals of Leicester NHS Trust, Glenfield Hospital, Groby Road, Leicester, LE3 9QP UK; 8 Institute of Applied Health Research, University of Birmingham, Edgbaston, Birmingham, B15 2TT UK; 9 REACH-HF Patient and Public Involvement Group, c/o Research, Development & Innovation, Royal Cornwall Hospitals NHS Trust, BIU, Knowledge Spa, Royal Cornwall Hospital, Truro, Cornwall TR1 3HD UK; 10 Sandwell & West Birmingham Hospitals NHS Trust, Sandwell General Hospital, Lyndon, West Bromwich, West Midlands B71 4HJ UK; 11 University of Exeter Medical School (Primary Care), Truro Campus, Knowledge Spa, Royal Cornwall Hospital, Truro, TR1 3HD UK

**Keywords:** Heart failure, Self-care intervention, Rehabilitation, Physical activity, Intervention mapping, Behaviour change

## Abstract

**Background:**

We aimed to establish the support needs of people with heart failure and their caregivers and develop an intervention to improve their health-related quality of life.

**Methods:**

We used intervention mapping to guide the development of our intervention. We identified “targets for change” by synthesising research evidence and international guidelines and consulting with patients, caregivers and health service providers. We then used behaviour change theory, expert opinion and a taxonomy of behaviour change techniques, to identify barriers to and facilitators of change and to match intervention strategies to each target. A patient and public involvement group helped to identify patient and caregiver needs, refine the intervention objectives and strategies and deliver training to the intervention facilitators. A feasibility study (ISRCTN25032672) involving 23 patients, 12 caregivers and seven trained facilitators at four sites assessed the feasibility and acceptability of the intervention and quality of delivery and generated ideas to help refine the intervention.

**Results:**

The Rehabilitation Enablement in Chronic Heart Failure (REACH-HF) intervention is a comprehensive self-care support programme comprising the “Heart Failure Manual”, a choice of two exercise programmes for patients, a “Family and Friends Resource” for caregivers, a “Progress Tracker” tool and a facilitator training course. The main targets for change are engaging in exercise training, monitoring for symptom deterioration, managing stress and anxiety, managing medications and understanding heart failure. Secondary targets include managing low mood and smoking cessation. The intervention is facilitated by trained healthcare professionals with specialist cardiac experience over 12 weeks, via home and telephone contacts. The feasibility study found high levels of satisfaction and engagement with the intervention from facilitators, patients and caregivers. Intervention fidelity analysis and stakeholder feedback suggested that there was room for improvement in several areas, especially in terms of addressing caregivers’ needs. The REACH-HF materials were revised accordingly.

**Conclusions:**

We have developed a comprehensive, evidence-informed, theoretically driven self-care and rehabilitation intervention that is grounded in the needs of patients and caregivers. A randomised controlled trial is underway to assess the effectiveness and cost-effectiveness of the REACH-HF intervention in people with heart failure and their caregivers.

**Electronic supplementary material:**

The online version of this article (doi:10.1186/s40814-016-0075-x) contains supplementary material, which is available to authorized users.

## Background

Heart failure is a complex and unpredictable condition which substantially affects the quality of life of over 26 million patients and their families worldwide [1]. It is associated with around 1–3 % of total healthcare expenditure in Western Europe, North America and Latin America, with hospitalisation being a key driver of costs [2, 3].

To manage heart failure effectively, patients need to engage in a number of self-care behaviours, including taking medications, monitoring symptoms, seeking help when required, eating and drinking healthily and managing depression [4–6]. In particular, improving and maintaining physical fitness can have a major impact on the ability of patients to engage in activities of daily living, such as preparing meals and using stairs. A recent Cochrane systematic review including 33 randomised trials in 4740 individuals with heart failure showed that cardiac rehabilitation based on exercise significantly reduces the overall risk of hospitalisation (relative risk 0.75) and of heart failure-specific hospitalisation (relative risk 0.61) as well as improving patient health-related quality of life [7]. Based on this and other high quality evidence, The American College of Cardiology/American Heart Association, European Society of Cardiology (ESC) and the National Institute for Health and Care Excellence (NICE) in the UK all recommend exercise-based cardiac rehabilitation and self-care as effective and safe adjuncts to the management of heart failure [4, 5, 8].

Nevertheless, practice surveys in the UK and Europe have shown that only around a sixth of people with heart failure are offered targeted (heart failure-specific) rehabilitation programmes [9, 10] and less than half of those offered cardiac rehabilitation attend [11]. Two key proposed reasons for such poor participation are that the majority of current rehabilitation services are hospital- or centre-based programmes and that they lack involvement from carers. Centre-based programmes pose problems of geographical accessibility, physical accessibility due to fatigue and potential co-morbidities [11], dislike of groups [12] and fitting participation in around work or domestic commitments [9].

Family members or caregivers can influence the self-care of people with heart failure [13, 14], and practice guidelines for heart failure recommend that caregivers are included in discussions about care [5]. Furthermore, the physical and mental health of caregivers may be affected by the demands of the caregiving role and this may affect their ability to offer support [15–17]. However, few trials of interventions for people with heart failure have involved caregivers [18].

The development of a home-based intervention to support self-care (including physical rehabilitation through exercise), which also includes a substantial caregiver support component, therefore has potential to enhance the current management of heart failure. In this paper, we describe the development and theoretical underpinnings of the Rehabilitation Enablement in Chronic Heart Failure (REACH-HF) self-care and rehabilitation intervention, which is designed to improve health-related quality of life in people with heart failure and their caregivers [19].

## Methods and results

### Framework for intervention development

Following the UK Medical Research Council guidance for developing complex healthcare interventions [20], we used a systematic, evidence-informed approach to develop the REACH-HF intervention. Our approach was based on intervention mapping, a six-step systematic framework for intervention development [21].

- Step 1: “needs assessment” to identify targets for change.
- Step 2: building matrices to “map” change objectives against determinants of the desired changes.
- Step 3: selection of appropriate behaviour change techniques and strategies to address each determinant identified in step 2.
- Step 4: production of detailed intervention and training materials.
- Step 5: anticipating adoption and implementation of the intervention.
- Step 6: plans for evaluation of processes and effects.

Intervention mapping was chosen as it is a well-established and widely used framework for development of health service interventions. It seeks to ground the intervention in the context and the population to be targeted, as well as the existing evidence base. It works backwards from the expected programme objectives (i.e. changes that need to happen in order to achieve the desired health outcomes), to identifying barriers to (and enablers of) achieving the objectives and then identifying intervention strategies that will facilitate change.

**Fig. 1:**
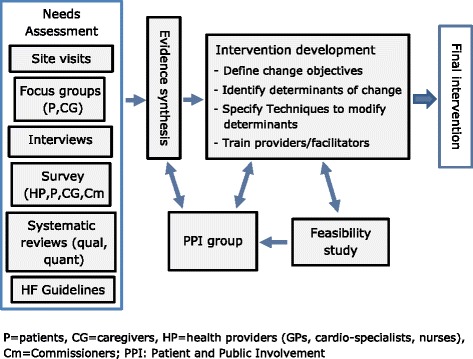
The intervention development process

A key element of the intervention development process was patient and public involvement (PPI) [22]. The REACH-HF programme has an active PPI group consisting of six people from Cornwall with a range of experiences of heart failure and three caregivers of people with heart failure. An overview of the intervention development process is provided in Fig. 1, and the following sections provide a summary of the first five steps of the intervention mapping process, as applied to developing the REACH-HF intervention.

### Step 1: needs assessment/identifying targets for change

#### Identification of needs

**Fig. 2:**
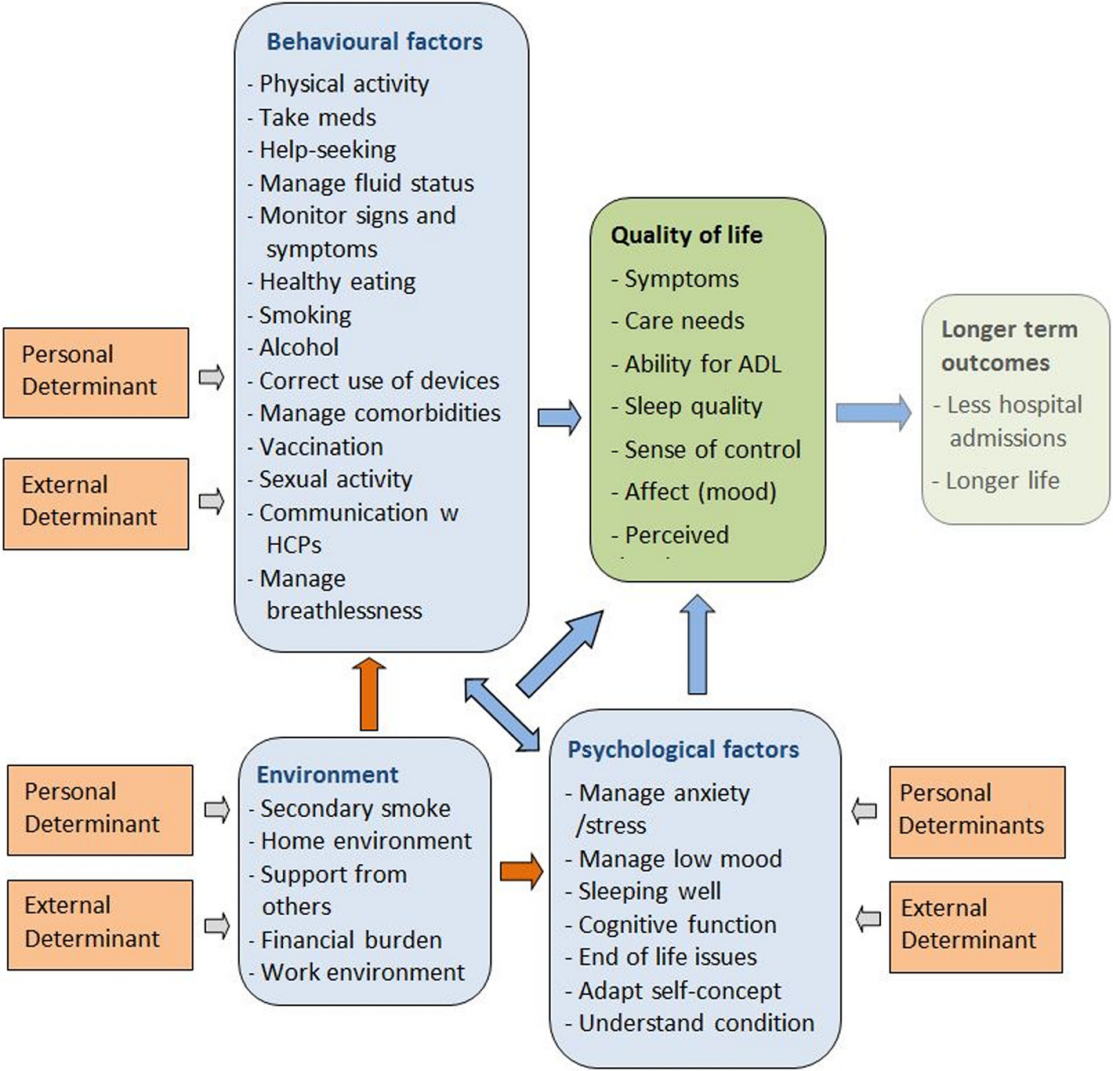
The REACH-HF causal model for the self-management of heart failure

The process began by assessing the needs of heart failure patients, caregivers and service providers. The aim was to summarise, as stated by Bartholomew et al. [21, p. 195] “what is and what is more desirable”. This included gathering information on the problem and its causes and on the target population, and developing a “causal model” outlining the main modifiable factors that might contribute to an improvement in quality of life for people with heart failure (Fig. 2).

Data sources included are provided in Table 1.

**Table 1:** Data sources and methods for needs assessment

Data type	Patients	Caregivers	Potential facilitators	Other health professionals and topic experts
Review and synthesis of qualitative literature	✓ [14]	✓ [14]	X	X
Systematic reviews and meta-analyses	✓ [23, 57–64]	X	X	X
Needs assessment survey	✓	X	✓	✓
Postal survey of NHS providers	X	X	X	✓ [9]
Site visits	X	X	✓	✓
Focus group interviews	✓	X	X	X
Face-to-face interviews and formal qualitative analysis	X	✓ [15]	X	X
Review of clinical guidelines	✓ [4, 5]	✓ [4, 5]	X	X
Expert opinion (meetings, focus groups)	X	X	✓	✓
Discussion with patient and public involvement group	✓	✓	X	X

✓ = data source used, X = not

Reviews of qualitative and quantitative literature provided a starting point for assessing the self-care support needs of patients and caregivers. An ongoing literature search (updated every 2–3 months) identified published reviews from 1994 onwards of self-care and rehabilitation interventions for people with heart failure. Given existing gaps in the literature, two de novo systematic reviews were undertaken by the project team: a meta-ethnographic synthesis of qualitative literature on the attitudes, beliefs and expectations of people with heart failure receiving cardiac rehabilitation [14] and a systematic review and meta-analysis of the efficacy and safety of cardiac rehabilitation in people with heart failure with preserved ejection fraction (HFPEF) [23]. Consultation with experts in the field, including the REACH-HF project management group, identified further epidemiological, social, behavioural and experimental evidence. We also reviewed national and international clinical guidelines for heart failure recommended by our project management group, including the ESC [4] and NICE practice guidelines [5]. The key recommendations on behaviour change, information needs, or other changes needed to improve the quality of life of patients or caregivers were extracted along with potential self-care strategies and potential determinants of such changes.

A number of systematic reviews and guidelines highlighted the importance of exercise-based rehabilitation as central elements in driving positive outcomes in heart failure [4, 5, 23, 24]. As a result, a specialist working subgroup of project team members (PD, SS, KJ, JA, CG, RT) met several times (along with extensive email interaction) to develop and refine the exercise and physical activity components of the intervention.

We conducted focus group interviews with two community-based heart failure support groups and attendees at a hospital-based rehabilitation class. Each group included 12 to 20 patients and 6 to 10 caregivers. The main topic areas were “coming to terms with heart failure” (including problems associated with the condition and how they were resolved); benefits of and barriers to exercise/physical activity; problems and solutions associated with taking medications; information and support needs; advice for family members or caregivers and how the REACH-HF intervention should be delivered.

To further elicit the views of key stakeholders, a needs assessment questionnaire was circulated to ten people with heart failure and 24 other “experts in the field” including two behavioural scientists, 14 specialist nurses (heart failure, cardiac rehabilitation and primary care cardiac nurses), two cardiologists, two GPs, two exercise physiologists with cardiac rehabilitation experience and two pharmacists. This was an opportunity sample based on contacts known to the REACH-HF project management group and people in the focus groups who had volunteered to complete the questionnaire. The questionnaire (Additional file 1) was designed to expand on the preceding literature reviewing and focus groups. It included questions about what outcomes were important for people with heart failure; self-care behaviours that should be targeted; information and support needs; suggested content and delivery formats and who might deliver the intervention. Respondents were also asked how the manual could be adapted for a range of users (including those with HFPEF).

The REACH-HF PPI group helped to design the topic guide for the focus group interviews, they completed and commented on the needs assessment survey and commented on summaries of information from the focus groups. The group met every 2 months throughout the 12-month needs assessment stage with additional e-mail and postal correspondence between meetings.

A qualitative research study involving face-to-face, semi-structured interviews with a purposive sample of 26 caregivers of people with heart failure with a range of gender, age and socio-economic status was conducted to specifically identify caregivers’ needs [15].

Understanding the context or community in which an intervention is delivered is another important aspect of needs assessment [21]. A member of the research team (WA) conducted site visits to heart failure treatment centres and a range of staff at four sites (Truro, York, Birmingham and Abergavenny) and administered a questionnaire on current service provision [25]. This identified existing strengths, relevant competencies and capacities of potential providers. In addition, heart failure specialist nurses, senior cardiac rehabilitation nurses and experts from the REACH-HF programme management group assessed the strengths and weaknesses of existing heart failure services at each study site.

Two team members (MC, CD) reviewed further literature to identify evidence on the effectiveness of relaxation and mindfulness interventions for people with heart failure (and other chronic illnesses) to inform the stress management component of the Heart Failure Manual. Finally (just prior to implementation in the feasibility study), a training needs questionnaire was sent to health professionals who had been selected to deliver the intervention, to assess their current state of knowledge/expertise with regard to key elements of the intervention. This was used to tailor the training course in step 4.

#### Analysis and integration of needs assessment data

A key challenge was to summarise and integrate the data and ideas from many diverse sources. We did this using a framework for mixed-mode evidence synthesis called Triangulation Protocol [26]. First, a thematic synthesis of the needs assessment documents and recordings was used to generate a “Needs Assessment” table (Additional file 2), which listed the key recommendations from each evidence component. We then considered where the recommendations from each source agreed (convergence), offered complementary information on the same issue (complementarity) or seemed to be contradictory (dissonance). Where there was dissonance, we resolved this through further discussion with the members of the project management and PPI groups (for example, some nurse respondents to our survey were not comfortable with patient self-titration of diuretics, although patients and guidelines suggested that this was acceptable with clear guidance for people who were willing and confident to take it on). The focus of the data synthesis was on identifying (a) targets for change and (b) modifiable determinants of the changes suggested. This analytic process was conducted separately for patients and caregivers.

The themes identified by the above synthesis were organised into a logic model [27] for the intervention (Fig. 2). This was developed by grouping the targets for change into broad themes (behavioural, environmental, social and psychological) and mapping them onto a generic causal modelling framework for intervention development (the PRECEDE model [21, 28]). It was acknowledged that environmental and contextual factors (e.g. home environment, social support networks) might affect health-related quality of life directly or indirectly (via interaction with behavioural or psychological factors).

#### Prioritisation and intervention focus

The targets for change (Table 2) were then prioritised through a process that included noting the level of agreement between stakeholders (from the needs assessment table); consultation with the project PPI group; and further discussion within the project team. We took into account the level of agreement between stakeholders, the strength of the evidence base and the potential for improving health-related patient quality of life. The highest priority targets for change (shown in dark grey in Table 2) were then grouped into the following five categories:

1. Engaging in exercise training to build (and maintain) cardiovascular fitness
2. Managing stress, breathlessness and anxiety
3. Heart failure symptom monitoring (and associated help-seeking), particularly in terms of managing fluid status
4. Taking prescribed medications
5. Understanding heart failure

**Table 2:**
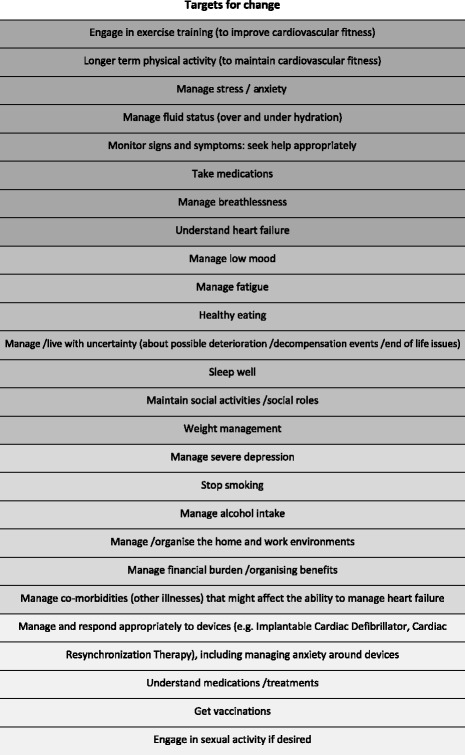
Developing the REACH Heart Failure Manual—targets for change

Key:

Full coverage (core topic and important for all)

Brief, needs-based intervention (topic important for some but not all patients)

Case management approach (topic important for some, but needing external input)

Information only (topic peripheral or of relatively minor importance in most cases)

Item 5 (understanding heart failure) was included following step 3 (below) as it was identified as a core determinant underpinning engagement with the first four targets for change. This is consistent with the “common sense model” of Leventhal et al., which posits that irrational behaviour is often based on misconceptions about the condition or mis-interpretation of symptoms (so it is logical to the individual) [29, 30]. For instance, in order for people to be motivated to increase physical activity, it is important for them to understand (and believe) the rationale that physical activity could have positive effects on heart failure symptoms/limitations due to heart failure.

The project management group and PPI group agreed that the above core priorities should receive strong, focused support from the intervention facilitator and that the intervention manual should contain interactive elements to support change in these areas (e.g. for exercise training, we included of a choice of a walking programme or chair-based exercise programme, as well as interactive tools for goal-setting and self-monitoring).

A second set of targets (the second shaded block in Table 2) were identified as important for some but not all patients (e.g. smoking cessation, healthy eating). It was agreed that these aspects should be assessed and (briefer) intervention from the facilitator provided if needed. The manual content for these targets provides information or tips on what to do if there is a problem associated with these areas (or if it becomes a problem) and (if appropriate) assessment tools to help assess the individual’s level of need.

A third set of targets, although important for some patients, were deemed to be outside the remit of the provider (e.g. management of severe depression), or possible to address through existing services (e.g. smoking cessation). It was agreed that these topics would be dealt with using a case-management approach. The REACH-HF intervention content for these topics primarily consists of information or tips, with self-assessment in some instances to facilitate recognition of the problem (e.g. for depression). The REACH-HF facilitator assesses the patient, briefly discusses the issues and may signpost the patient to an appropriate health professional or organisation if a problem is identified. She/he may continue to monitor progress with this issue during the intervention period.

A fourth set of targets were categorised as peripheral or minor topics such as vaccination. For these topics, the patient is assessed, given some information and signposted if needed to further agencies, or information (e.g. websites).

#### Caregiver targets for change and prioritisation

The core priorities for the caregiver resource were:

1. To facilitate improvement in quality of life for the person with heart failure by helping them to achieve the core priorities for change for patients (as above).
2. To improve quality of life for caregivers by acting to maintain their own health and well-being.

The target of “understanding heart failure” was also felt to be of core importance in underpinning engagement with the above targets. The targets for change for caregivers that emerged from the needs assessment process are shown in Table 3. A focus group with four caregivers was conducted to prioritise the targets in the same way as described for the patient manual.

**Table 3:**
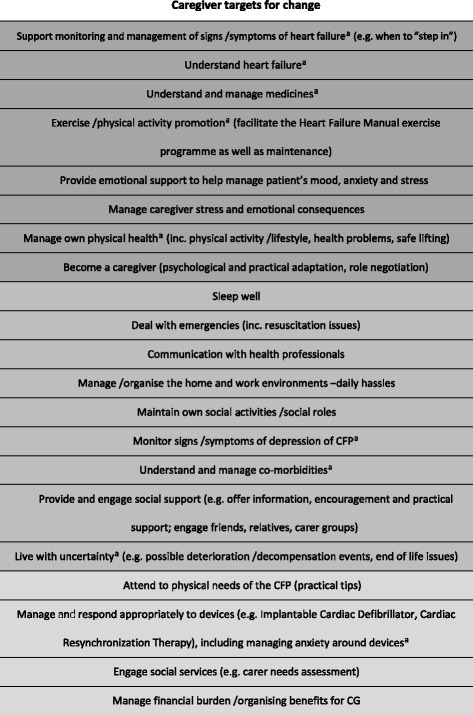
Developing the REACH-HF Caregiver Resource—targets for change

*CG* caregiver, *CFP* cared for person

^a^These self-care issues are also dealt with in the Heart Failure Manual, and relevant sections are referenced from the caregiver resource

Key:

Full coverage (core topic and important for all)

Brief, needs-based intervention (topic important for some but not all patients)

Case management approach (topic important for some, but needing external input)

Information only (topic peripheral or of relatively minor importance in most cases)

### Step 2: specifying performance objectives/identifying determinants of change

The behavioural, environmental, social and psychological targets for change resulting from needs assessment (Tables 2 and 3) were broken down into more proximal “performance objectives”. Performance objectives are statements of who needs to change and what behaviours or thought processes need to be changed (and in what circumstances) to achieve each target [21].

For each performance objective, modifiable determinants of change were identified using several parallel methods:

- Existing evidence (e.g. process evaluations in rehabilitation studies).
- Theories of behaviour change and psychological adaptation [14, 29, 31–35]
- Evidence identified during the needs assessment stage (e.g. qualitative data, needs assessment questionnaire)
- A structured 1-day workshop with a panel of experts in the field (two exercise/rehabilitation specialists, two cardiac specialist nurses, two GPs with cardiac special interest, two cardiologists, three behavioural scientists)
- Similar structured workshops (three separate 2-h sessions) with the PPI group. The consultation workshops focused on the “core priority” change targets

In the workshops, the “core priority” targets for change and their associated performance objectives were presented to the expert panel and the PPI group. For each performance objective, the panel was asked the following:

1. What will help people to achieve this target?
2. What will stop people achieving this target? What will get in the way?
3. How could we help people to overcome any barriers and achieve this target?

A facilitated group discussion resulted in a list of modifiable determinants (barriers and facilitators) relating to each objective.

The performance objectives and determinants were then used to construct a set of “mapping matrices” or tables. The first two columns in Table 4 show the performance objective and determinants for the objective of “Engage in exercise training sessions two to three times per week”. Separate matrices of performance objectives and determinants were constructed for the caregiver intervention.

**Table 4:** Section of intervention mapping matrix for the performance objective “engage in exercise training” (NB: this is only a selection from the full intervention map for the wider change target “engaging in exercise training and physical activity to build (and maintain) cardiovascular fitness)”

Performance objective	Modifiable determinants	Change techniques	Strategies
1. Engage in exercise training sessions two to three times per week: NB: Exercises designed to improve cardiovascular fitness, improve efficiency of movement and enhance the ability to perform functional activities associated with daily living (by building and maintaining muscle strength and prevention of muscle shortening).	Perceived importance/treatment efficacy Illness perceptions	Provide information on consequences^a^ Provide information on illness identity, timeline, causes, consequences and control to build a functional understanding/illness model (how HF works and how exercise affects HF)^b^ Simultaneous self-monitoring of behaviour and symptoms^b^ Prompt intention formation^a^ Motivational interviewing^a^	Manual text on benefits of PA/fitness in relation to HF symptoms (M). Self-monitoring of symptoms (inc. mood, sleep quality) alongside progress with exercise to help build associations between exercise and health/learning from experience (M). Reflections by facilitator on these associations (F). Discussion of existing knowledge about how heart failure works and how self-care actions affect symptoms, mood/stress and quality of life (F). Assessment of barriers to activity and tailored support/encouragement using MI techniques (F).
	Time	Time management^a^	Assessment of barriers to activity and tailored support (F), including a time management activity in the manual (M).
	Support from others	Plan social support (informational, emotional, practical)^a^	Assessment of barriers to activity and tailored support (F), including exploration of social support. Engage caregiver in a supporting role, with encouragement, planning and practical help (F). Provide caregiver with Family and Friends manual (CGM).
	Physical capacity	Individual tailoring of exercise level to current fitness^b^	Set starting level to match existing capacity (based on incremental shuttle walk test (F).
	Confidence (self-efficacy)	Set graded tasks (graded efficacy and capacity building)^a^ Prompt-specific goal setting^a^ Prompt barrier identification (and problem solving)^a^ Prompt review of behavioural goals^a^ Motivational interviewing^a^	Multi-level DVD of graded exercises to demonstrate suitable exercises (M). Walking programme As an alternative to the DVD. Facilitation of use of action-planning and problem-solving tools in the manual and Progress Tracker (F, M). Regular review of progress and increasing goals for level/duration of exercise, when existing level becomes easy (F). Exploring and addressing barriers through use of MI techniques (F).
	Enjoyment	Offer choice of options for exercise (to address enjoyment)^b^ Simultaneous self-monitoring of behaviour and mood^b^	Patient to choose between DVD programme or walking programme (or a mixture). Self-monitoring of mood (and sleep quality) alongside progress with exercise to help build associations between exercise and positive mood/learning from experience (M). Reflections by facilitator on these associations (F).

The other performance objectives (not shown) were “2. Safely build up intensity/type of exercise as fitness improves to achieve a ‘basic level of fitness’; 3. Engage in a maintenance’ exercise regime at least twice weekly once a basic level of fitness has been achieved; 4. Monitor activity levels and maintain at a level that maintains fitness and quality of life, but does not lead to frequent bouts of exhaustion; 5. Restart the activity regime at an appropriate point following setbacks (e.g. a period of illness); 6. Learn how to assess level of exertion to exercise at the right level; 7. Learn how to assess level of breathlessness and take appropriate action before it gets out of control”

*M* manual content, *F* facilitator task, *CGM* caregiver manual

^a^Techniques listed in the Abraham and Michie taxonomy [36]

^b^Techniques not listed in the Abraham and Michie taxonomy [36]

### Step 3: specification of change techniques and strategies

Step 3 of the intervention mapping process involved the selection of change techniques (e.g. behaviour change techniques, psychological intervention techniques) targeting each of the determinants of change identified in step 2. In addition to expert opinion and experience (e.g. several strategies were recommended by the stakeholder groups in step 2, based on their own experiences), this work drew on an existing taxonomy of behaviour change techniques [36] and the expertise of the REACH-HF collaborators in developing disease management programmes and cardiac rehabilitation programmes to identify potentially successful strategies for heart failure patients and their caregivers. For example, delivery methods and strategies (including techniques for stress management) from an existing evidence-based self-care support intervention for myocardial infarction (the Heart Manual) [37, 38] were employed in the REACH-HF manual. The PPI group were asked about the strategies they had found to be successful and reviewed the selected change strategies (and the final programme materials) to ensure they were likely to be feasible and acceptable for patients and caregivers.

It is worth noting that the behaviour change taxonomy was used as a source of ideas rather than a definitive/exclusive set of options, and a number of novel, non-taxonomy techniques were also used (these are indicated in the table footnotes). The change techniques and strategies for their delivery were added (as separate columns) to the intervention matrix. Table 4 shows an extract of the intervention matrix for exercise training. This illustrates that, in order to accomplish the performance objective of engaging in exercise training two to three times per week, the barrier of “having insufficient time” must be addressed. This was achieved (partly) by using time management techniques. We opted to provide a choice of exercise options, as this was desired by the patient representatives and it was expected that having a choice would improve enjoyment of the exercise and adherence [39, 40]. Further extracts from the intervention mapping tables for the other core targets are presented as Additional file 3. If readers are interested in replicating our approach or using our materials, the full intervention and training materials are available on request via the lead author (CG) or the study co-CI (RT).

#### Underlying theory/processes of change

Alongside the specification of intervention techniques, considering the theoretical underpinnings (the way in which the selected techniques are supposed to address determinants and promote change) can be useful in terms of (a) guiding the choice of intervention techniques (where there are multiple options), (b) helping to structure and organise the intervention materials (for example, the underlying theory may imply a preferred sequencing of techniques) and (c) helping to inform process evaluations (to test and refine the intervention mechanisms going forward) [20, 27]. In this case, different challenges (barriers) were identified for different change targets and so the resulting intervention is multi-theoretical. Despite this, several common theoretical processes for supporting the targeted changes in behaviour and psychological processes were identified, and these are outlined in Table 5. In summary, the intervention drew on several theoretical perspectives, but key principles included building understanding of the condition to provide a rationale for change (Leventhal’s Common Sense Model [29]) such as how physical fitness affects heart failure symptoms); building intrinsic motivation and promoting autonomy (Self-Determination Theory [31]); promoting adaptation to living with heart failure and adopting an active rather than passive approach to coping [14, 41]; and encouraging learning from experience through engagement in self-regulation activities (Control Theory [42]). The elements aimed at managing stress and anxiety used psychological intervention processes based on cognitive behaviour therapy [43] and mindfulness therapy [44, 45].

**Table 5:** Theory and processes for supporting behaviour change in the REACH-HF intervention

Process (and theoretical basis)	Key features and intervention facilitation techniques
ACTIVE PATIENT INVOLVEMENT (motivational interviewing [46]/Self-Determination Theory [31])	The facilitator should encourage the participant to be actively involved in the consultation. The idea is to maximise the participant’s autonomy as the main agent of change, developing intrinsic rather than extrinsic motivation. However, the consultation should be guided. Empathy-building skills (Open questions, Affirmation, Reflective listening, Summaries) and individual tailoring should be used throughout the consultations. Reflective listening may be used to direct the conversation or highlight key strengths or barriers. A collaborative/shared decision-making style is appropriate, and the facilitator may share his/her own expertise and ideas. The Ask-Tell-Discuss technique should be used to exchange information (e.g. to address misconceptions, or offer helpful new information). Overall, the participant should be increasingly empowered to take control of her/his self-care behaviour. Interactions should be encouraging, respectful and non-judgemental. The interaction should also be *individually tailored* to the patient’s specific information needs, beliefs, skills and priorities.
ASSESSING THE PATIENT’S CURRENT SITUATION AND NEEDS (motivational interviewing [46], individual tailoring [65])	The facilitator should use patient-centred communication techniques (as above) which may include the Ask-Tell-Discuss and open-ended questions to explore the patient’s current situation. This should include all of the following: identify and discuss the most important issue currently for the patient, how well are they managing their fluids, how appropriately are they using medications, is there any obvious immediate clinical need, how much stress or anxiety do they have, how much physical activity are they doing and what other concerns or questions they may have.
FORMULATING AN INDIVIDUALISED TREATMENT PLAN (Self-Regulation/Control Theory [42], individual tailoring [65])	The facilitator should use patient-centred communication techniques (as above) to formulate an appropriate treatment plan based on the patient’s current situation (as assessed above). The treatment plan will be staged over time, aiming to work on a few topics initially and introducing other elements as the programme continues. This should be set up as an experiment to see how feasible the proposed actions are and whether they help the patient’s situation. An element of guiding to ensure the inclusion of clinical priorities (e.g. medication issues, exercise) as well as patient priorities may be appropriate. The facilitator and participant should formulate a specific written action plan (using the template in the Progress Tracker) for exercise-training based on a choice of the two REACH-HF exercise-training programmes. The patient and caregiver should be ‘signposted’ to relevant sections of the manual. The facilitator may also employ some problem-solving techniques at this stage to pre-empt and address potential problems.
BUILDING THE PATIENT’S UNDERSTANDING OF HEART FAILURE/THEIR SITUATION (Leventhal’s common sense model [29], theories of illness adaptation [14, 41])	The facilitator should elicit the patient’s and caregiver’s current understanding of heart failure and seek to build their “illness model” in terms of understanding the identity, causes, consequences, cure/control options and timeline associated with the condition. This process may take several weeks and should be reinforced as the programme progresses. Facilitators will signpost the patient and caregiver to relevant sections of the manual, including the “Understanding Heart Failure” section and use patient-centred communication techniques (as above) to elicit and build understanding. The Ask-Tell-Discuss technique and reflective listening will be used to exchange information to reinforce elements of the patient’s understanding that predispose positive self-care behaviours (e.g. understanding the link between physical fitness and symptoms of HF). The facilitator should seek to reframe negative attitudes and exchange information to address misconceptions or address important gaps in understanding. Learning should be reflected on/reinforced at subsequent sessions.
SUPPORTING SELF-REGULATION SKILLS (Self-Regulation/Control Theory [42], relapse prevention [66], theories of illness adaptation [14, 41])	The facilitator should discuss and encourage the use of the “Progress Tracker” workbook in the HF Manual to keep track of progress and as a way of recording and addressing any problems in completing the activities and any benefits that might be associated with the planned activities. At subsequent meetings, the facilitator and participant should review progress with all planned changes to exercise/physical activity and other self-care activities. The facilitator should reinforce and reflect on any successes. The participant and facilitator should discuss any setbacks, encourage identification and problem-solving of barriers to self-care and the patient’s plans should be revised accordingly. Reframing should be used to normalise setbacks and see them as an opportunity to learn from experience (trial and error) rather than as failures. Problem-solving should use Open questions, Affirmation, Reflective listening, Summaries (OARS) and information exchange (Ask-Tell-Discuss) techniques to identify barriers and explore ways to overcome them. Problem-solving may specifically focus on issues of connectedness (social influences, involvement of others in supporting activities) and long-term sustainability, or on breaking the problem down into more manageable chunks.
ADDRESSING EMOTIONAL CONSEQUENCES OF HEART FAILURE (cognitive behavioural therapy [43], mindfulness [45], theories of illness adaptation [14, 41])	The facilitator should help the patient to recognise and address any significant stress, anxiety, anger or depression that is related to having heart failure. S/he should seek to normalise such feelings and help the patient to access and facilitate use of the cognitive behavioural therapy techniques and stress management techniques contained within the manual. If depression, anxiety or other emotional problems are severe, a referral to appropriate clinical services should be facilitated.
CAREGIVER INVOLVEMENT (if applicable) (literature on caregiver needs [15])	The facilitator should engage the caregiver as much as possible as a co-facilitator of the intervention. S/he should tailor the intervention to work with the caregiver’s abilities and availability. Person-centred counselling techniques (OARS) should be used for caregiver assessment and to exchange information to build the caregiver’s understanding of the situation and to help them recognise and manage their own health needs including mental health, physical health and social needs. He/she should facilitate a conversation between the patient and the caregiver to agree to their roles and responsibilities and how these might change if the patient’s condition declines. Attention should be given to the caregiver’s needs and concerns about being a caregiver/providing care as well as those of the patient. The facilitator should help the caregiver to recognise and address any significant stress, anxiety, anger or depression that is related to supporting someone with heart failure and facilitate the use of the cognitive behavioural therapy techniques and stress management techniques contained within the manual as needed. This includes facilitating a referral for a carer’s assessment if the caregiver wishes, plus referral to other relevant care services as appropriate. The facilitator should help the caregiver to prioritise and look after his/her own health and well-being.
BRINGING THE PROGRAMME TO A CLOSE (Leventhal’s common sense model [29], theories of illness adaptation [14, 41], Self-Regulation/Control Theory [42], relapse prevention [66])	Progress should be consolidated and reinforced. Plans for long-term sustainability of activities and strategies learned for managing heart failure should be discussed. The facilitator will review progress since the start of the intervention and reinforce what has been learnt. Useful strategies that were helpful should be identified. Plans to stay well/prevent relapse should be discussed as well as “cues for action” and plans to revisit the manual in the future. The facilitator will discuss plans to sustain any new activities, identifying any potential problems and coping strategies to overcome these. The possibility of good and bad days should be discussed and normalised.

### Step 4: production of detailed intervention and training materials

The outputs from the first three stages of the intervention mapping process were used to generate detailed intervention materials and a training course for facilitators. The four main REACH-HF intervention elements were:

- The Heart Failure Manual: A written self-help resource for use by patients and their caregivers. The resource includes a choice of two structured exercise programmes: A chair-based exercise DVD (developed by one member of the research team (PD) and colleagues specifically for people with heart failure) with seven levels of progressively increasing intensity which guides participants through exercises designed to build cardiovascular fitness and to strengthen muscles to facilitate activities of daily living; and a progressive walking-training programme based on increasing walk duration and intensity over time to build cardiovascular fitness (and leg muscle strength). The starting level (for the DVD) or walking time (for the walking programme) was set based on results from an incremental shuttle walk test (using a table which allows matching of the metabolic equivalent (MET) value of the patient’s individual test results against the MET values for different levels of the training activities). The manual also includes a CD for relaxation and breathing control exercises from the existing Heart Manual [38].
- The Progress Tracker: An interactive booklet designed to facilitate learning from experience/over time and the building of understanding about how self-care activities impact on symptoms, emotional well-being and quality of life, through practice, self-monitoring of progress and (facilitated) problem solving.
- The Family and Friends Resource: a manual for use by caregivers. This aims to increase caregiver understanding and skills both for helping the person with heart failure and for looking after their own physical and mental well-being. The resource is divided into three main sections: 1. Supporting the patient’s self-management of heart failure (“Providing Support”), 2. Caring for the caregiver (“Being a caregiver”) and 3. Practical advice including mobilising social support, accessing benefits and other formal and voluntary support (“Getting Help”).
- A training course for facilitators. A training manual/syllabus for a 3-day training course for REACH-HF intervention facilitators was developed. Facilitators were defined as professionals with experience in cardiac rehabilitation or cardiac nursing. The facilitation role is crucial to the success of the REACH-HF programme. As well as being the main delivery process, it enables tailoring of the REACH-HF intervention resources to the individual needs of patients and their caregivers. The course includes the theory and process of facilitation (building rapport using patient-centred counselling techniques [46], empowerment and support of self-management, building understanding of the condition [29]); using behaviour change techniques; techniques for managing stress and anxiety; contents of the manual; supporting exercise and physical activity using the intervention materials; facilitation of the Family and Friends Resource and medical/nursing issues. The training was linked by three case studies of heart failure patients and opportunities to practice facilitation techniques and to problem-solve potentially difficult situations. Additional file 4 outlines the overall facilitation process.

The PPI group commented on the above materials in terms of both format and content. For instance, members of the group tried out the chair-based exercise DVD. They agreed that this would be a helpful component especially for patients with co-morbidities that limit mobility. The group also indicated that we should include the ability to mix and match exercise programmes if patients wished to do this. The PPI chair (KP) co-delivered content at all three training days. A set of quotes or “patient voices” from patients and caregivers in the PPI group and from qualitative interviews were also incorporated into the written resources to help illustrate key points.

#### Intervention delivery

The project management and PPI groups agreed that the REACH-HF intervention should be considered for patients with a confirmed diagnosis of heart failure with reduced ejection fraction in the last 5 years, who have been clinically stable for at least 2 weeks and who are deemed suitable for exercise [47]. There was less evidence to inform adaptations of the intervention for people with HFPEF. However, the project team agreed that the Heart Failure Manual may benefit patients from this group as they have similar symptoms and there is emerging evidence demonstrating the benefits of exercise in patients with HFPEF [23, 48]. Based on existing cardiac rehabilitation practice, 12 weeks was considered an appropriate duration for delivery with a minimum of three face-to-face contacts with a facilitator (plus telephone contacts) during this time. The face-to-face contacts are delivered in the patient’s home.

Facilitators were trained to deliver the intervention using patient-centred counselling techniques [46] and to individually tailor/target intervention components to the needs and priorities of the patient (and the caregiver).

### Step 5: anticipating adoption and implementation issues

Following ethical approval, a feasibility study (ISRCTN25032672) was conducted to assess the feasibility and acceptability of the intervention, extract ideas to help refine the intervention in advance of the main trial, and assess the quality of intervention delivery.

The REACH-HF intervention was delivered to 23 patients (and 12 caregivers) by seven trained facilitators at four sites (Cornwall, Abergavenny, Birmingham and York). Process data to help assess feasibility, acceptability and quality of intervention delivery (intervention fidelity) was collected from multiple sources, including recordings of intervention sessions, contact report forms, satisfaction questionnaires and interviews with both patients and caregivers. Both patients and caregivers gave written, informed consent.

**Fig. 3:**
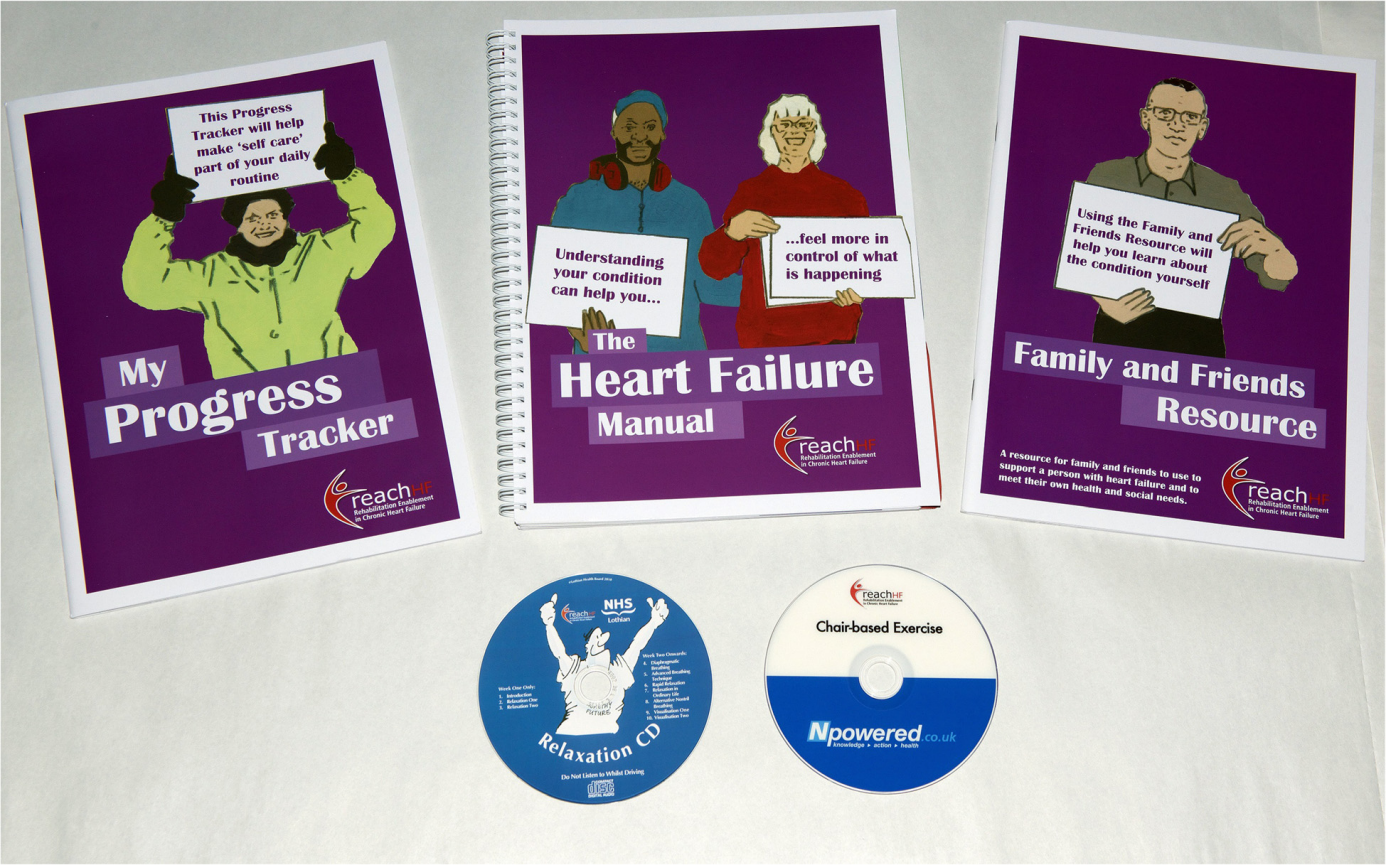
The REACH-HF intervention materials

A summary of the findings is available in Additional file 5. We found that there was a high level of satisfaction with the intervention (and of the research evaluation procedures) from facilitators, patients and caregivers and good engagement with the intervention by both patients and caregivers. A number of ideas for improving the text of the Heart Failure Manual and for improving the training were identified such as changing the name of the original “Caregiver Resource” to the “Family and Friends Resource” to promote engagement with the intervention (many co-habitees did not identify themselves as a “caregiver”). Analysis of the quality of intervention delivery, based on applying a checklist to recordings of all the consultations for 18 cases, suggested that the components of the intervention were mostly delivered as intended and with high quality. However, there was room for improvement in terms of addressing caregiver health and emotional health. No adverse patient safety issues were identified. As a result, the Heart Failure Manual (including the Family and Friends Resource and the Progress Tracker) and training course were substantially revised (Fig. 3 shows the revised materials).

## Discussion

This paper describes the development of an evidence-based exercise rehabilitation and self-care intervention facilitated by health professionals for heart failure patients and their caregivers—the Rehabilitation Enablement in Chronic Heart Failure (REACH-HF) intervention. The development process described in this paper was consistent with the MRC framework for developing complex healthcare interventions [20]. Intervention mapping gave a clear structure and process for developing the intervention and the associated training programme for intervention facilitators. The process took into account the needs of a range of stakeholders including heart failure patients, their caregivers, health professionals, potential facilitators and healthcare commissioners.

The construction of a causal model as part of the intervention mapping method (Fig. 2) was useful as a framework for integrating the needs identified and defining the intervention’s “targets for change”. However, as reported by other studies that have used intervention mapping to develop complex healthcare interventions [49–51], the overall process was time-consuming and resource-intensive.

Patient and public involvement was an integral and important part of the whole process central in the development of the REACH-HF intervention and was a particular strength of the research. The PPI group exemplified the importance of producing an intervention that is tailored to individual needs based on a diverse range of patient backgrounds, knowledge levels and severity of heart failure.

It is not clear if the same results would have been obtained using other frameworks such as the Behaviour Change Wheel [52], or the implementation of change model [53]. However, the processes that would have been involved in using these alternative frameworks are very similar to our chosen approach, including assessing the needs of the population of interest, establishing a clear “behavioural diagnosis”, mapping existing service provision, identifying barriers and facilitators of change and identifying change strategies to address barriers and boost facilitating influences. Some frameworks recommend using a checklist of Theoretical Domains [54] to assist the identification of potential barriers/enablers of change (i.e. to identify change processes) [35, 55]. Intervention mapping does not preclude this, but in our case we identified barriers/enablers using a more “bottom-up” stakeholder-oriented approach (deriving them from extensive qualitative and quantitative assessment of the needs of service users, carers and service providers). We also ensured that common theoretical themes were identified and used to inform the structuring and delivery of our intervention.

One way in which we adapted intervention mapping was to use it to plan changes in psychological processes such as managing stress/anxiety and addressing low mood, as opposed to targeting only changes in behaviour. The determinants identified here went beyond those covered by the Theoretical Domains Framework. These included dynamic influences such as downward spirals in depression (e.g. low mood leads to negative thoughts, social withdrawal and negative physical effects on physical health, which leads to lower mood) and more psychological determinants of change such as “persistent negative thoughts (e.g. intrusive thoughts about death, being a burden to others)” or “avoidance (due to thoughts of death or due to not wanting to appear weak or vulnerable)”.

### Strengths and limitations

This is one of the few studies to describe in detail the theoretical and evidentiary basis, intervention techniques and strategies for an intervention for promoting the quality of life of people with heart failure and their caregivers. The main limitation was the complexity of the process, which affects replicability and requires considerable resources. Despite a transparent audit trail and documentation of all the processes involved, it is unlikely that a different team of collaborators using the same methods would have produced exactly the same intervention. The implementation could have taken a number of different forms, as “judgement calls”, decisions and selection of appropriate methods or theoretical approaches were required at many stages during the process. Although having a panel of experts helped to ensure that judgement calls involved multiple stakeholders and decisions were based on either evidence or appropriate expertise, there was often no clear “best solution” and a different group of experts may have come up with different solutions. Intervention development therefore remains as much an art as a science. It depends on the individual expertise, experience, instincts and knowledge of the team (in this case, the multi-disciplinary REACH-HF investigators and the PPI group) as well as on team dynamics and collective decision-making.

### Implications and future directions

Further research is now needed to assess the effectiveness and cost-effectiveness of the REACH-HF intervention. A multi-site, fully powered randomised trial (ISRCTN86234930) in patients with reduced ejection fraction heart failure [56] and their caregiver and a single-centre pilot randomised trial in patients with HFPEF (ISRCTN78539530) and their caregivers are currently in progress. This includes a mixed methods process evaluation to assess mechanisms of change, based on testing key elements of the logic model in Fig. 2. Beyond this, further research might include (a) adaptation and evaluation of the intervention into a digital format, with more emphasis on remotely delivered or online support; (b) implementation research about how the REACH-HF programme (if effective) could be integrated with existing health service models/infrastructure; (c) assessment of the impact on effectiveness of using (more resource-intensive) formative feedback to enhance the training of facilitators; (d) the impact of such interventions on longer term outcomes such as mortality and hospital admissions.

## Conclusions

Intervention mapping, along with strong service user involvement, was a resource-intensive, but rigorous, method, which allowed the development of a comprehensive, evidence-informed, theoretically driven facilitated self-care and rehabilitation intervention that is grounded in the needs of heart failure patients, caregivers and service providers.

## Abbreviations

DVD, digital versatile disk; HFPEF, heart failure with preserved ejection fraction; PPI, patient and public involvement; ESC, European Society of Cardiology; REACH-HF, Rehabilitation Enablement in Chronic Heart Failure

## Additional files

Additional file 1: Needs assessment questionnaire. (PDF 552 kb)
Additional file 2: REACH-HF Needs Assessment Summary. (PDF 492 kb)
Additional file 3: Extracts from intervention maps for stress management, medication management and managing symptoms. (PDF 267 kb)
Additional file 4: The REACH-HF Facilitation Process. (PDF 280 kb)
Additional file 5: Rehabilitation Enablement in Chronic Heart Failure (REACH-HF) Feasibility Study Report. (PDF 563 kb)
