# Supplementary material for: Optimising self-care support for people with heart failure and their caregivers: development of the Rehabilitation Enablement in Chronic Heart Failure (REACH-HF) intervention using intervention mapping
Source: Pilot Feasibility Stud. 2016 Aug 2;2:37. doi: 10.1186/s40814-016-0075-x (PMC5153822; doi:10.1186/s40814-016-0075-x)
Supplement: Additional file 1: — Needs assessment questionnaire. (PDF 552 kb) [file 40814_2016_75_MOESM1_ESM.pdf]

Additional File 1: Needs assessment questionnaire to elicit initial ideas on the content, scope and delivery methods for the proposed REACH-HF Heart Failure Manual.

Please read the following and **add your own ideas** where indicated ….

1. The range of people that the Heart Failure Manual could be offered to

Current ideas:
- We will offer the Heart Failure Manual to people of any age with a confirmed diagnosis of heart failure, identified from GP or hospital records (Echo-cardiography showing a Left Ventricular Ejection Fraction less than 45% and natriuretic peptide levels (BNP) of 100pg/l or less, or NTProBNP of 400pg/ml or more).

- We also intend to design materials for people with HFPEF (Heart Failure with Preserved Ejection Fraction)

- The manual will be offered only after discharge from any recent hospital stay.

What people should **not** be offered a Heart Failure Manual?
Current ideas:
- People with terminal illness (e.g. terminal cancer), including people with severe heart failure who are entering palliative care services.

- People with communication difficulties or mental health problems that would prevent engagement with the service - although we can still provide the manual to carers in these circumstances.

Your ideas: *Please add any further ideas here …*

2. What kinds of information and support do people with Heart Failure need to help them to manage or 'self-care' for their condition effectively? Self-care of heart failure can be defined as actions aimed at maintaining physical stability, avoidance of behaviour that can worsen the condition, and detection of the early symptoms of deterioration (ESC guidelines, 2008).

Please add any ideas that you feel are missing in the empty space at the bottom of the table. Please tell us whether each item should be A) excluded B) included as part of the "Core Content" of the manual (i.e. if you think it is an essential issue for every patient) or C) included in an 'optional ' section of the manual (i.e. this issue will be covered only if needed or desired by the individual patient) .

**Content that the Heart Failure Manual should include**	**Should this content be ...**<br>A: excluded<br>B: included as essential content for **every** heart failure patient<br>C: included as "additional information" depending if it is important for a particular person?		
People with heart failure should be helped to develop a clear understanding of their situation. What causes heart failure, what does it mean for the individual (possible risks and consequences, what you can and can't do and how different actions might affect your symptoms) and what can be done to best manage the situation? Common mis-conceptions should be identified and addressed. (Expert opinion, REACH review of patient experiences)	A	B	C
People's reactions and responses to the development of heart failure vary considerably. Explaining and helping people to move through the 'five stage process' of disruption, conception, reaction, response and assimilation might be helpful here. (REACH review of patient experiences)	A	B	C
Encourage engagement in regular moderate daily activity (including both aerobic and muscle-strengthening activities). Patients should be re-assured and comfortable about engaging in physical activity (ESC[1] guidance)<br>A recent ESC Position Statement (2011) advises at least 20 min, a minimum of three times a week aiming to achieve modest breathlessness during exertion.	A	B	C
Encourage engagement in physical activity training to improve mobility and the efficiency of movement and enhance the ability to perform functional activities associated with daily living (e.g. stair climbing, getting out of chairs). This may need to be individually tailored to build from the patient's initial level of ability. (Expert opinion)<br>The principle here is that the heart may not be able to improve its output but the muscles can, with the correct form of exercise make more efficient use of the available oxygen. (Expert opinion)	A	B	C

[1] European Society of Cardiology

**Content that the Heart Failure Manual should include**	**Should this content be ...**<br>A: excluded<br>B: included as essential content for **every** heart failure patient<br>C: included as "additional information" depending if it is important for a particular person?		
Taking the medications in the right way /as prescribed. This includes understanding what each medication is for and when to take them and also what effects or side effects to expect. (ESC[1])	A	B	C
Addressing concerns about medications, other treatments /interventions and their side effects. (Expert opinion)	A	B	C
The need for the dosage or type of medications to be changed over time should also be understood. (REACH review of patient experiences)	A	B	C
How to achieve clear and appropriate communication with healthcare professionals (ESC)	A	B	C
Engaging support from others (e.g. friends, family) to support self-care (ESC)<br>This should include ideas about information support, emotional support and practical support. Consideration of how to manage if you are socially isolated may also be relevant here. (Expert opinion)	A	B	C
Patients should learn to recognize the symptoms of deterioration and take appropriate action such as increasing the dose of prescribed diuretic medications and /or contacting the healthcare team. (ESC)<br>Agreeing a strategy for managing medications with the nurse-facilitator may be important here, including knowing when to adjust doses or when to consult with the nurse or GP. (Expert opinion)	A	B	C
Patients should weigh themselves on a regular basis to monitor weight change, preferably as part of a regular daily routine. In the case of a sudden unexpected weight gain of 2 kg or more in 3 days, patients may increase their diuretic dose and should alert the healthcare team. (ESC)	A	B	C

**Content that the Heart Failure Manual should include**	**Should this content be ...**<br>A: excluded<br>B: included as essential content for **every** heart failure patient<br>C: included as "additional information" depending if it is important for a particular person?		
Restricting salt in the diet for people with 'symptomatic heart failure' to prevent fluid retention (ESC. NB: there is no guidance on specific levels, but excessive intake should be avoided. The Mayo clinic recommends <2.0g per day or <1.5g /day for Afro-Caribbeans)<br>NB: This recommendation needs further investigation, as it would seem that restricting salt intake may be harmful in some cases. It may be that we do not have enough evidence to make any specific recommendation about which patients should restrict salt intake and at what levels.	A	B	C
People with severe symptoms and where this has been identified by a health professional as being important should restrict fluid intake or take other measures to manage their fluid status. (ESC)	A	B	C
Alcohol intake should be limited to 10–20 g/day (1–2 glasses of wine/day, 2 units for women, 3 for men). (ESC)<br>Patients with alcohol induced cardiomyopathy should stop drinking alcohol altogether. (Expert opinion)	A	B	C
Make changes to diet to reduce weight if Body Mass Index is more than 30Kg/m2. However, this is not recommended for people with evidence of cachexia (involuntary weight loss /body wasting). (ESC)	A	B	C
Basic advice about what constitutes a healthy diet for people with heart failure and maintaining a healthy body weight. We should refer to sources of relevant recipes online and elsewhere. (Expert opinion)	A	B	C

**Content that the Heart Failure Manual should include**	**Should this content be ...**<br>A: excluded<br>B: included as essential content for **every** heart failure patient<br>C: included as "additional information" depending if it is important for a particular person?		
Advice on smoking cessation (for those who smoke). (ESC). Avoidance of secondary smoking. (Expert opinion)<br>It is suggested that brief information on the risks and consequences is provided, and if desired, referral by the facilitator to appropriate smoking cessation services. (Expert opinion)	A	B	C
Pneumoccocal vaccination and annual influenza vaccination (as long as no contra-indications). (ESC)	A	B	C
Be re-assured about of the safety of engaging in sexual activity, if desired. Explore concerns about sexual activity. Be aware of and able to use possible strategies for prevention of dyspnoea (breathing difficulties) and chest pain during sexual activity (e.g. nitroglycerine tablets). (ESC)	A	B	C
Women with heart failure should (if applicable) be aware of the potential risks associated with pregnancy and should discuss the use of contraceptives and /or planned pregnancy with a physician in order to take informed decisions. (ESC)	A	B	C
High altitudes (1500 m or more) and travel to very hot and humid destinations should be discouraged for symptomatic patients. Planned travel should be discussed with the healthcare team. (ESC, ESC Position Statement, 2011)	A	B	C
Sleep apnoea (breathing problems during sleep) should be recognised and steps taken to manage it. This may involve weight loss in severely overweight persons, smoking cessation, abstinence from alcohol, or use of CPAP treatment (breathing masks) if needed (ESC). Elevation of the head during sleep, and changing of eating and timing of diuretic medication may also be considered (Mayo Clinic)	A	B	C

**Content that the Heart Failure Manual should include**	**Should this content be ...**<br>A: excluded<br>B: included as essential content for **every** heart failure patient<br>C: included as “additional information” depending if it is important for a particular person?		
Manage the emotional impact of living with heart failure (ESC).<br>The aim here should be to help the individual to adapt his or her lifestyle, life goals and expectations to the challenge presented by heart failure. The individual should be able to move from being a person disrupted by heart failure to being a person living with and coping with heart failure. The sense of attaining some form of ‘good-enough’ control, a sense of ‘safety’ and acceptance of the limitations and the inherent uncertainty of heart failure seem to be important here. This may include finding (or re-defining) the individual’s role within the home and in the wider world and in relation to friends /family. (REACH systematic review of patient experiences)<br>An analysis of the individual’s past, present and possible future social roles might be relevant – acknowledging any current discrepancies /loss of roles and thinking about what is possible in the future. (Expert opinion)	A	B	C
Recognising and taking steps to manage depression, anxiety and other mental health aspects also seem important. (Expert opinion)<br>This may include engaging in cognitive-behavioural therapies, engaging support from friends /family, use of medications or use of stress-relieving techniques. (Expert opinion)	A	B	C
Managing co-morbidities (other illnesses) that might affect the ability to manage heart failure. Especially mobility limitations, diabetes, COPD, other heart conditions. (ESC and expert opinion)<br>This may include strategies for remembering to take medications regularly (e.g. pre-loaded pill boxes) and discussion of options with a pharmacist. (Expert opinion)	A	B	C

**Content that the Heart Failure Manual should include**	**Should this content be ...**<br>A: excluded<br>B: included as essential content for **every** heart failure patient<br>C: included as "additional information" depending if it is important for a particular person?		
Manage breathing difficulties /breathlessness and stress /anxiety using breathing control /relaxation techniques. (Expert opinion)	A	B	C
Managing the consequences of cognitive impairment /memory problems (e.g. remembering to take medications) (Expert opinion)	A	B	C
Self-monitoring of symptoms, energy levels and other important outcomes to identify possible problems and solutions. (Expert opinion)	A	B	C
Self-monitoring of blood pressure if blood pressure is high. (Expert opinion)	A	B	C
Engagement in problem solving and /or goal-setting to facilitate the above self-care behaviours (Expert opinion)	A	B	C
Use of home telemetry (remote monitoring of health indicators and access to advice via computer links to appropriate specialists) where available. (Expert opinion)	A	B	C
Correct use of devices (e.g. Implantable Cardiac Defibrillator, Cardiac Resynchronization Therapy). Understanding how to manage and respond to device alarms, where appropriate (Expert opinion)	A	B	C
Returning to work. (Expert opinion)	A	B	C
Planning and pacing yourself. Practical solutions, for managing activities of daily living, for example taking small loads of washing to the line. (Expert opinion)	A	B	C
Negotiation of the role of any caregivers /supporters. (Expert opinion)	A	B	C
Information about benefits that may be available and how to claim them. (Expert opinion)	A	B	C

Content that the Heart Failure Manual should include	Should this content be ...<br>A: excluded<br>B: included as essential content for **every** heart failure patient<br>C: included as "additional information" depending if it is important for a particular person?	
**Please insert your own ideas below**		
		B	C
		B	C
		B	C
		B	C
		B	C
		B	C
		B	C

2b. How would the inclusion of people with Heart Failure with Preserved Ejection Fraction (HFPEF) affect the proposed content above? Which of the above elements will need to be different or not needed for those with HFPEF?

Your ideas: *Please add your ideas here....*

3. What are the key messages for people with Heart Failure? What messages do you believe would be useful in helping them to cope with /manage their Heart Failure?)

Current ideas:
- Importance of regular exercise /activity
- Ensure that medications are used in the best way to enable you to function positively
- Heart failure can usually be managed at home. Hope is important here.
- You are still an important and valued person.
- Support from health care professionals including after the initial support programme has finished.
- Ask from (your heart failure nurse, GP) for help if you are not coping.

Your ideas: *Please add your ideas here....*

4. What information do carers or relatives need and what behaviours should we try to encourage for these caregivers?

**Ideas for carer behaviours that the Heart Failure Manual should encourage**	**Should this content be**<br>A: excluded<br>B: included in the "Carers Manual"	
Caregivers should learn to recognize the symptoms of deterioration and help to take appropriate action such as increasing the prescribed diuretic dose and/or contacting the healthcare team. (ESC, Expert opinion)	A	B
Caregivers should help to manage the emotional impact of living with heart failure (ESC). This may include discussing problems, helping their loved one to feel valued, or help with the use of stress-relieving techniques. (Expert opinion)	A	B
Negotiation of the caregiver role / what support is required to self-manage the problem (e.g. managing medication, weighing, emotional support/encouragement, observing). (Expert opinion)	A	B
Caregivers should look after their own health, including ways to manage the emotional impact on themselves. (Expert opinion)	A	B

Caregivers should help to ensure good communication and a good working relationship with health professionals. (Expert opinion)	A	B
Caregivers should help the person they care for to understand and engage with all the other self-care behaviours listed above. (Expert opinion)	A	B
Care but don't smother! Support patients to be as independent as possible and find their maximum potential. (Expert opinion)	A	B
Information about benefits and respite care. (Expert opinion)	A	B
**Please insert your own ideas below**		

5. Under what conditions should we advise patients to consult with their GP or Heart Failure Manual nurse?

Current ideas:

- Weight gain that does not respond to adjustment of diuretic dosage (JW)
- When they feel the symptoms or worries are hindering their confidence to effectively self-manage.
- Increase in breathlessness or fatigue, especially at rest
- If they feel the plan isn't working and that the patients QoL is deteriorating
- A guidance booklet or card (optional) and a page in the manual formatted as a 'traffic light' system may be appropriate here
- If patient is consistently feeling ''down or in a low -mood''

- If they notice a step change in their ability to do basic daily activities
- If patient has been unable to take medication

Your ideas: *Please add your ideas here....*

6b. In what circumstances should we advise patients or their carers to seek emergency care?

Current ideas:
- Loss of consciousness (CG)
- Chest pains or sudden onset of palpitations (potential arrhythmia)
- If ICD (implanted defibrillator) shocks are applied for patients with these devices
- A guidance page or (optional) booklet formatted as a 'traffic light' system may be appropriate here

Your ideas: *Please add your ideas here....*

7. Are there any topics that we should NOT cover in the Heart Failure manual (e.g. too specialist or contentious, or potentially dangerous?)
Current ideas:
- Detailed coverage of palliative care (end of life) issues? (Expert opinion)

Your ideas: *Please add your ideas here....*

8. What is a 'good outcome' for a person with Heart Failure?

Current ideas:
- Reduced mortality /longer life
- Reduced morbidity (less symptoms, better Quality of Life, lower frequency of fluid retention problems, less breathlessness, increased exercise capacity)
- Reduced need for hospitalisation
- Reduced progression of cardiac health indicators (occurrence or progression of myocardial damage, myocardial remodelling
- Reduced costs of care
- Improved efficiency i.e. more or the same level of work for less effort
- Improved ability to live independently /to achieve activities of daily living

Your ideas: *Please add your ideas here....*

9. What range of delivery staff might deliver /facilitate the Heart Failure Manual intervention? (CRNs, PNs, other?)

Current ideas:
- Cardiac rehabilitation nurses, with additional training in heart failure treatment
- Practice nurses with appropriate training (this is contentious with some experts agreeing and others not)
- Heart failure specialist nurses
- Given the level of co-morbidity and the likely range of initial abilities, there should also be access to a specialist in adapting exercise e.g. physiotherapist or exercise specialist working in cardiac or pulmonary rehab services.
- Other specialists that it may be sensible to be able to refer on to might include dietitians and clinical psychologists

Your ideas: *Please add your ideas here....*

10. We don't want to re-invent the wheel, so what sources of information are relevant here that we can draw on ? For example, existing guidance, content of existing intervention programmes, existing patient information resources.

Current ideas:
- ESC Position Statement on Self-Care of Heart Failure. European Journal of Heart Failure 2011 Feb 1;13(2):115-26.
- BACR (2007)
- ESC
- NICE (CG5, 2010) and SIGN guidelines on heart failure
- The existing Heart Manual.
- Service Specification for Cardiac Rehabilitation (Dept of Health, 2010)
- NICE CG43 on obesity; CG48 on physical activity; PH1,5,10 on smoking
- Other Physical activity guidelines (ACPICR, 2009; ACSM (2010); AACVPR (2010)
- Guidance on patient education and supporting behaviour change : BACR, 2007; SIGN, 2002; NICE 2007
- Guidance on Cardiovascular Risk Factor management: NICE CG48; NICE CG34; NICE CG66; NICE - - CG87; NICE CG15; NICE CG67
- Davies et al 2010
- NICE CG 108
- www.heartfailurematters.org (ESC patient information website)
- BHF heart failure manual

Your ideas: *Please add your ideas here....*

11. Because people with heart failure have different needs for support, we are proposing to have two elements to our manual. One part of the manual will be the same for everyone and will deal with all the "Core Issues" – the things that everyone needs to know about and do to manage their condition. The second part will contain "Tailored Information" that only *some people* will need – for example there is no point giving you information about smoking if you don't smoke, or about managing your implanted defibrillator if you don't have one. So, the question that arises, is "How should we present the Tailored Information?" There are four choices here:-

A. Give all the information to everyone and tell you which chapters are relevant to you.

B. Give you a separate booklet for each piece of Tailored Information that is relevant to you.

C. Have the nurse pick out the right information chapters for you and put them into a ring binder, so that you only have only the information that is relevant to you and it is all gathered together in one place.

D. Provide website references so that you can look up the additional information yourself online.

Which option would you prefer? **A** **B** **C** **D** *(please circle one)*

12. For clinicians: What way should the nurse-facilitator training be conducted? Face to face; e-learning; mixture of both; other?

13. How might we need to adapt the manual for different demographic and ethnic /cultural groups?

Your ideas: *Please add your ideas here....*

14. A major component of rehabilitation for people with heart failure is to engage with as much physical activity as possible at a sensible / achievable level. The needs and abilities of individual patients will vary widely however and so we would like to explore the possibility of providing access to an appropriately qualified exercise specialist. So, would it be realistic to offer people with heart failure …

A. Group-based activity sessions in the community, led by a qualified (Stage 3 or above) exercise specialist?

B. 1-2 Individual sessions in the patient's home to design a home-based exercise programme to fit the individual's needs (based on information supplied by the nurse who is facilitating the Heart Failure Manual intervention)?

C. A choice of the above options?

D. None of the above

Which options are realistic? **A** **B** **C** **D** *(please circle one or more)*

Ideas for the content of a 'manual' for Heart Failure: Questions for service user advisors

Please read the following and **add your own ideas** where indicated ….

2. Who is the Heart Failure Manual for?

Current ideas: - We will offer the Heart Failure Manual to people of any age with a confirmed diagnosis of heart failure

What people should **not** be offered a Heart Failure Manual?

Current ideas:

- People who are entering palliative (end of life) care services.

- People with communication difficulties or mental health problems that would prevent engagement with the service - although we can still provide the manual to carers in these circumstances.

Your ideas: *Please add any further ideas here …*

2. What are the key messages for people with Heart Failure? Please feel free to highlight any that you think are particularly good or cross out any you think are wrong

Current ideas:

- Regular exercise /activity is important to keep you fit enough to get out and about and do the things you want to do in your life
- Getting too little physical activity gets you into a downward spiral (you lose fitness and can do less and then you lose more fitness ...etc).
- Building up your physical activity /exercise to a manageable level helps you to maximise your potential (as you gain fitness, you can do more and get on with your life more easily)
- However, don't over-do it - doing too much can make you worn out the next day
- Ensure that medications are used in the best way to enable you to function positively
- Heart failure can usually be managed at home.
- Hope is important.
- You are still an important and valued person.
- You are not alone /help is available for coping with the condition even after the initial care programme has finished.
- Ask for help (from your heart failure nurse, or GP) if you are not coping.
- Don't give up!
- It is important to keep up a positive mental attitude

- Do not ignore fluid build up or other changes in symptoms
- Heart failure disrupts your life but it is possible to learn to live with it
- Healthy eating helps
- No smoking – this is the worst possible thing you can do as far as your heart is concerned

- A number of patients don't understand what has happened. Often, they see it as a sudden event rather than the result of a decline. They therefore don't think it can be repaired or improved, when often it can. Understanding the condition helps them to understand what the treatment is trying to achieve and why.
- Self monitoring and early reporting of change in symptoms is key to managing the condition
- Patient empowerment is important – what they can do to be in control of their condition i.e monitoring weights, fluid restriction when appropriate, maintaining fitness etc.
- HF can't be cured, but there are ways to manage it to have as good a quality of life as you can
- We need to say clearly what are the early warning signs (when action is needed by the patient)
- We need to say clearly what are the urgent warning signs (when emergency care is needed)
- Heart failure is a serious condition (I know hope is important but it also should not be taken lightly!)

Your ideas: *Please add your ideas here....*

2. What is a 'good outcome' for a person with Heart Failure?

Current ideas:
- Longer life
- Less symptoms, better Quality of Life, lower frequency of fluid retention problems, less breathlessness, increased exercise capacity
- Reduced need for hospitalisation and NHS service use
- Improved efficiency i.e. more or the same amount of things achieved with less effort
- Improved ability to live independently /to achieve activities of daily living
- Return to work
- Feeling happy within themselves i.e. at ease /not stressed or emotional

Your ideas: *Please add your ideas here....*

3. What kinds of information and support do people with Heart Failure need to help them to manage or 'self-care' for their condition effectively? Self-care of heart failure can be defined as actions aimed at maintaining physical stability, avoidance of behaviour that can worsen the condition, and detection of the early symptoms of deterioration.

Content that the Heart Failure Manual should include	Your comments
People with heart failure should be helped to develop a clear understanding of their situation. What causes heart failure, what it means for the individual (possible risks and consequences, what you can and can't do and what can be done to best manage the situation? Common mis-conceptions should be identified and addressed.	
People's reactions and responses to the development of heart failure vary considerably. Explaining and helping people to move through the 'five stage process' might be helpful here. The five stages of adaptation to heart failure are:<br>1. Disruption – initial chaos and disruption to normal life patterns and emotions following diagnosis<br>2. Making sense of the situation<br>3. Reaction – this can range from denial (ignoring the problem) to positive engagement (doing things to address the problem)<br>4. Response - making plans about what to do to best manage the problem, trying out new ideas and strategies<br>5. Assimilation – learning how to live with the condition and to maximise quality of life, accepting and working around any remaining limitations.	
Encourage engagement in regular moderate daily activity (including both aerobic and muscle-strengthening activities). Patients should be re-assured and comfortable about engaging in physical activity<br>A recent European guideline (2011) advises at least 20 min, a minimum of three times a week aiming to achieve modest breathlessness during exertion.	

Content that the Heart Failure Manual should include	Your comments
Encourage engagement in physical activity training to improve mobility and the efficiency of movement. This should aim to work on the ability to perform functional activities associated with daily living (e.g. stair climbing, getting out of chairs). This may need to be individually tailored to build from the patient's initial situation.	
Taking the medications in the right way /as prescribed. This includes understanding what each medication is for and when to take them and also what effects or side effects to expect.	
Addressing concerns about medications, other treatments /interventions and their side effects.	
The need for the dosage or type of medications to be changed over time should also be understood.	
How to achieve clear and appropriate communication with healthcare professionals	
Engaging support from others (e.g. friends, family) to support self-care<br>This should include ideas about information support, emotional support and practical support.	
Patients should learn to recognize the symptoms of deterioration and take appropriate action such as increasing the dose of prescribed diuretic medications and /or contacting the healthcare team.	
Patients should weigh themselves on a regular basis to monitor weight change, preferably as part of a regular daily routine. In the case of a sudden unexpected weight gain of 2 kg or more in 3 days, patients may increase their dose of diuretic medication and should alert the healthcare team.	
Alcohol intake should be limited to 10–20 g/day (1–2 glasses of wine/day, 2 units for women, 3 for men).<br>Patients with alcohol induced cardiomyopathy should stop drinking alcohol altogether.	
Basic advice about what constitutes a healthy diet for people with heart failure and maintaining a healthy body weight. We should refer to sources of relevant recipes online and elsewhere.	

Content that the Heart Failure Manual should include	Your comments
Advice on smoking cessation (for those who smoke). Avoidance of secondary smoking.	
Encouraging annual flu vaccinations.	
Be re-assured about of the safety of engaging in sexual activity, if desired. Explore concerns about sexual activity. Be aware of and able to use possible strategies for prevention of breathing difficulties and chest pain during sexual activity (e.g. nitroglycerine tablets).	
Women with heart failure should (if applicable) be aware of the potential risks associated with pregnancy and should discuss the use of contraceptives and /or planned pregnancy with a physician in order to take informed decisions.	
High altitudes (1500 m or more) and travel to very hot and humid destinations should be discussed with the healthcare team.	
Breathing problems during sleep should be recognised and steps taken to manage it. This may involve weight loss in severely overweight persons, smoking cessation, abstinence from alcohol, or use of CPAP treatment (breathing masks) if needed. Elevation of the head during sleep, and changing of eating and timing of diuretic medication may also be considered.	
Manage the emotional impact of living with heart failure:<br>The aim here should be to help the individual to adapt his or her lifestyle, life goals and expectations to the challenge presented by heart failure. The individual should be able to move from being a person disrupted by heart failure to being a person living with and coping with heart failure. The sense of attaining some form of ‘good-enough’ control, a sense of ‘safety’ and acceptance of the limitations and the inherent uncertainty of heart failure may be important here. This may include finding (or re-defining) the individual’s role within the home and in the wider world and in relation to friends /family.	
Recognising and taking steps to recognise and manage depression, anxiety and other mental health problems that may occur.	

Content that the Heart Failure Manual should include	Your comments
Managing other illnesses that might affect the ability to manage heart failure. Especially mobility limitations or joint problems, diabetes, COPD and other heart conditions.<br>This may include strategies for remembering to take medications regularly (e.g. pre-loaded pill boxes) and discussion of options with a pharmacist.	
Manage breathing difficulties /breathlessness and stress /anxiety using breathing control /relaxation techniques.	
Managing memory problems (e.g. remembering to take medications)	
Self-monitoring of symptoms, energy levels and other important outcomes to help to identify possible problems and solutions.	
Self-monitoring of blood pressure if it is high.	
Returning to work.	
Planning and pacing yourself. Practical solutions, for managing activities of daily living, for example taking small loads of washing to the line.	
Negotiation of the role of any caregivers /supporters.	
Information about benefits that may be available and how to claim them.	
**Please insert your own ideas below**	

4. What information do carers or relatives need and what behaviours should we try to encourage for these caregivers?

Ideas for carer behaviours that the Heart Failure Manual should encourage	Your comments
Caregivers should help the person they care for to understand and engage with all the self-care behaviours listed above.	
Caregivers should learn to recognize the symptoms of deterioration and help to take appropriate action such as increasing medication dose or seeking medical help.	
Caregivers should help to manage the emotional impact of living with heart failure (ESC). This may include discussing problems, helping their loved one to feel valued, or help with the use of stress-relieving techniques	
Working with your loved one to decide what support is required (e.g. managing medication, encouragement, observing) and what responses might be unhelpful.	
Caregivers should look after their own health, including ways to manage the emotional impact on themselves.	
Caregivers should help to ensure good communication and a good working relationship with health professionals.	
Care but don't smother! Support patients to be as independent as possible and find their maximum potential	
Information about benefits and respite care.	
**Please insert your own ideas below**	
