# Supplementary material for: Optimising self-care support for people with heart failure and their caregivers: development of the Rehabilitation Enablement in Chronic Heart Failure (REACH-HF) intervention using intervention mapping
Source: Pilot Feasibility Stud. 2016 Aug 2;2:37. doi: 10.1186/s40814-016-0075-x (PMC5153822; doi:10.1186/s40814-016-0075-x)
Supplement: Additional file 2: — REACH-HF Needs Assessment Summary. (PDF 492 kb) [file 40814_2016_75_MOESM2_ESM.pdf]

Additional File 2: REACH-HF Needs Assessment Summary

1. Patient needs (and objectives for optimal self-care)

a qualitative literature review of the attitudes, beliefs and expectations of people with HF receiving CR [45]

a systematic review and metaanalysis of CR in people with HFPEF[21]

Scaffolding questionnaire responses

A summary of the Support group /focus group interviews

National and international guidelines on heart failure treatment

2. Commissioner /health professional needs

National postal survey

Scaffolding questionnaire responses

Comments and ideas from co-applicant stakeholders (expert opinion)

3. Service provider needs (nurse facilitators)

Scaffolding questionnaire responses

Comments and ideas from co-applicant stakeholders (expert opinion)

To map existing service provision (the context we are going into), WA conducted site visits and meetings with service providers at each study site, as well as asking for responses to a questionnaire on current service provision and summarised this in a report.

4. Caregiver needs

Scaffolding questionnaire responses

Qualitative interviews with caregivers (WP1B)

National and international guidelines on heart failure treatment (minimal data)

5. Expert group meetings and workshops

A one-day expert panel workshop was held in Birmingham. This was used to refine the suggested programme objectives and identify barriers and facilitators of change using small group (groups of 3-4) discussions among the panel members.

A specialist working group was established to develop the PA component and this had three formal meetings, several teleconferences and ongoing email exchanges to refine the objectives, determinants and intervention strategies further and to produce the final specifications for the exercise (walking training or CBE) component, initial exercise capacity and safety assessment procedures and the manual text for this component.

Synthesis

We thematically analysed the summary reports /findings from all the above data sources to draw out some preliminary programme objectives (below) and then we used intervention mapping matrices to develop detailed strategies and intervention content.

Table 1: Programme objectives identified by the Needs Assessment process

**Programme objectives** (behl, psychl, envl outcomes to be targeted)	**Nurses**	**Patients /patient groups**	**Other**	**Guidance / evidence**	**QoL domain addressed (MLHFQ)**
**Engage with the programme**	Patient empowerment is important – what they can do to be in control of their condition i.e monitoring weights, fluid restriction when appropriate etc. (1. HF Nurse 2. HF /CR Specialist Nurse)<br><br>Patients who refuse rehab are offered it again at a later date. This is appropriate for	Post diagnosis, patients are often in shock and not ready to take on information. (Caregiver interviews)	It is self-evident that low engagement will lead to low effectiveness. So we need to maximise engagement with the programme. CG<br><br>Some patients may have strong denial /minimisation-of-the-problem issues at this stage, we may need to sell it to them a bit to get these people initially engaged. Minimisation is usually based on fear so need to provide re-assurance and messages of hope and efficacy. (HPsych).	It is recommended that patients with heart failure are enrolled in a multidisciplinary-care management programme to reduce the risk of heart failure hospitalization. (ESC 2012)	All domains (based on logic /assuming the intervention as a whole is effective, then increased engagement across all domains will increase effectiveness across all domains)

**Programme objectives** (behl, psychl, envl outcomes to be targeted)	**Nurses**	**Patients /patient groups**	**Other**	**Guidance / evidence**	**QoL domain addressed (MLHFQ)**
	us too as people have to be ready to face up to life with heart failure.(CRN)				
**Physical Activity**	May need to be individualised and a generic statement [e.g. 20 min 3x per week] may not be appropriate (StQ-HF N)<br>Think we need to increase the level building up (to at least 5 sessions per week 20-30 mins) as well as teaching people to self regulate in relation to their Borg RPE	Energy conservation /pacing (FG)<br>Learn how to assess your level of breathlessness and to be able to exercise at the right level /and to slow down before it gets out of control (FG)<br>The idea of teaching people to recognise their ventilatory threshold /other indicator of “exercising at the	Not just adherence to a set exercise regime, but being active as part of everyday life: climbing stairs, getting out and about, house work, walking the dog. (HPsychx2)<br>Energy conservation /pacing (PAS, HPsych)<br>At least 5 sessions per week 20-30 mins (Mayo clinic)<br>A key principle here is that the heart may not be able to improve its output but the muscles can, with the correct form of exercise make more efficient use of the available oxygen. (SCM-PAS, StQ)<br>One of the main advantages of the Heart Manual is that it is provides support for those that do not like	Supervised group exercise-based rehab programme designed for patients with<br>HF. (CG108)<br>Ensure patient is stable with no condition (e.g. uncontrolled hypertension) or device that would preclude PA. (CG108)<br>Regular aerobic exercise should be encouraged to improve functional capacity and symptoms. (ESC 2012)<br>At least 20 min, a minimum of three times	Should facilitate Activities of Daily Living (e.g. getting out and about, returning to work)<br>ESC 2012 cites grade A evidence linking PA to HF morbidity, HRQoL and hospital admissions.

**Programme objectives** (behl, psychl, envl outcomes to be targeted)	**Nurses**	**Patients /patient groups**	**Other**	**Guidance / evidence**	**QoL domain addressed (MLHFQ)**
	scores. (StQ-HF Nurse)<br><br>Important to bring the negative effects of over activity into the discussion (StQ-CRSN)	right level /when to slow down" was well received (HPsych /FG)<br><br>Need to be able to recognise symptoms leading up to exhaustion (FG)<br><br>Lists of ideas would be useful here (FG)<br><br>Unmonitored exercise at home should not be encouraged. Need to cover questions like: How long should I exercise? Should I be breathless? How energetic should I be? Should my heart be racing? Should	group work and are unlikely to turn up for group based rehab. (HMO expert)<br><br>Patients need to have confidence in exercising alone too, walking the dog, climbing stairs – things they are likely to be doing regularly as part of their normal routine and often alone.(HMO Expert)<br><br>Pacing of exercises that are done safely can also provide confidence. This is all part of the cognitive behavioural model. (HMO Expert)<br><br>Groups give support and reduce isolation. On the other hand, they can exert unintended peer pressure – "he's doing better than me" – and some people hate exercising in public, hence I think a choice is needed. A third possibility is an exercise video at various levels. This would also address variation in reading ability. (Pharmacist)<br><br>The opportunity to share experiences	a week aiming to achieve modest breathlessness during exertion.(ESC Pos St 2011)<br><br>Understand the benefits of exercise (HF Assoc, ESC 2012 Tab27)<br><br>Perform exercise training regularly. (HF Assoc, ESC 2012 Tab27)<br><br>Be reassured and comfortable about physical activity. (HF Assoc, ESC 2012 Tab27)	

**Programme objectives** (behl, psychl, envl outcomes to be targeted)	**Nurses**	**Patients /patient groups**	**Other**	**Guidance / evidence**	**QoL domain addressed (MLHFQ)**
		I be sweating? (StQ Patient)<br><br>Patient Focus Group expressed a strong preference for group based sessions due to the ability to share experiences with other people in the same situation and Supervision to ensure that exercise levels were safe (Patient FG).<br><br>Group-based exercise in the community /near the patient's home has the following benefits: Professional staff on site;	could be achieved through a support group instead. (HPsych)<br><br>Some kind of 'fitness to exercise' assessment may be needed to ensure that the patient is safe to exercise (SWGPA). The 6-minute walk test is sometimes used in hospital settings to provide an objective indicator of exercise capacity (ESC 2012).<br><br>**Birmingham workshop (expert opinion) …**<br><br>Overcome psychological barriers<br><br>Manage expectations<br><br>Education to include that some patients may need to drop a level or start at a much lower level after a period of illness.<br><br>Those completing a high dose level must build in rest periods between each session – 3 sessions spread through the week.		

**Programme objectives** (behl, psychl, envl outcomes to be targeted)	**Nurses**	**Patients /patient groups**	**Other**	**Guidance / evidence**	**QoL domain addressed (MLHFQ)**
		encouragement by staff and other patients; Opportunity for Monitoring progress (e.g. BP); No opportunity to cheat! (Patient)			
**Healthy eating**	Healthy eating, no smoking (HF Nurse)	Avoid excessive fluid intake: fluid restriction of 1.5–2 L/day may be considered in patients with severe heart failure to relieve symptoms and congestion. Restriction of hypotonic fluids may improve hyponatraemia. Routine fluid restriction in all patients with mild	Basic advice about what constitutes a healthy diet for people with heart failure and maintaining a healthy body weight. (StQ-HPsych)	Make changes to diet to reduce weight if Body Mass Index is more than 30Kg/m2. However, this is not recommended for people with evidence of cachexia (body wasting). (ESC, 2008?)	

**Programme objectives** (behl, psychl, envl outcomes to be targeted)	**Nurses**	**Patients /patient groups**	**Other**	**Guidance / evidence**	**QoL domain addressed (MLHFQ)**
		to moderate symptoms is probably not of benefit. Weight-based fluid restriction (30 mL/kg body weight, 35 mL/kg if body weight >85 kg) may cause less thirst. (HF Assoc, in ESC 2012, Tab27)<br><br>Monitor and prevent malnutrition. HF Assoc, in ESC 2012, Tab27)<br><br>Eat healthily and keep a healthy weight (HF Assoc, in ESC 2012, Tab27)			
		Need to be able to recognise	Managing fatigue /pacing is an important day-to-day challenge.		

**Programme objectives** (behl, psychl, envl outcomes to be targeted)	**Nurses**	**Patients /patient groups**	**Other**	**Guidance / evidence**	**QoL domain addressed (MLHFQ)**
**Manage fatigue**		symptoms leading up to exhaustion and take early action to prevent it (FG)<br><br>You have good days and bad days, so need alternative plans for the bad days. (Patient FG and PPI group)	Need to understand and adjust to the fact that people typically have 'good days and bad days' (REACH expert group workshop)<br><br>Planning and pacing yourself. Practical solutions, for managing activities of daily living, for example taking small loads of washing to the line. (StQ: Mixed sources give 81% approval for this idea)		
**Smoking**	Healthy eating, no smoking (HF Nurse)	Stop smoking and/or taking illicit drugs (HF Assoc, in ESC 2012, Tab27)		Advice on smoking cessation (for those who smoke). Avoidance of secondary smoking. (ESC,2008?)	
**Alcohol**		Modest intake of alcohol: abstinence is recommended in patients with		Alcohol intake should be limited to 10–20 g/day (1–2 glasses of wine/day, 2 units for women, 3 for men).	

**Programme objectives** (behl, psychl, envl outcomes to be targeted)	**Nurses**	**Patients /patient groups**	**Other**	**Guidance / evidence**	**QoL domain addressed (MLHFQ)**
		alcohol-induced cardiomyopathy. Otherwise, normal alcohol guidelines apply (2 units per day in men or 1 unit per day in women). 1 unit is 10 mL of pure alcohol (e.g. 1 glass of wine, 1/2 pint of beer, 1 measure of spirit) (HF Assoc, in ESC 2012, Tab27)		Patients with alcohol induced cardiomyopathy should stop drinking alcohol altogether. (ESC, 2008?)	
**Stress**		Provide psychosocial support to patients and family and/or caregivers. HF Assoc	Manage the emotional impact of living with heart failure: (StQ-HPsych)		
**Vaccination**			Approval 80% (and 5% for as-needed section on this). (StQ mixed	Check status and offer if needed annual flu,	

**Programme objectives** (behl, psychl, envl outcomes to be targeted)	**Nurses**	**Patients /patient groups**	**Other**	**Guidance / evidence**	**QoL domain addressed (MLHFQ)**
			sources)	and pneumococcal vaccination, as long as no contra-indications. (CG108)<br><br>Receive immunization against influenza and pneumococcal disease (HF Assoc /ESC 2012 Tab27)	
**Depression**		Provide psychosocial support to patients and family and/or caregivers. (HF Assoc)<br><br>Learn about treatment options if appropriate (HF Assoc /ESC 2012 Tab27)<br><br>Understand that depressive symptoms are	Recognising and taking steps to manage depression, anxiety and other mental health aspects. (StQ-HPsych)	Consider diagnosis. CG108<br><br>Reassess once heart failure has stabilised. CG108<br><br>Consider the risks and benefits of drug and other treatments. CG108<br><br>**The nurses' role here may be simply to identify and refer.**	

**Programme objectives** (behl, psychl, envl outcomes to be targeted)	**Nurses**	**Patients /patient groups**	**Other**	**Guidance / evidence**	**QoL domain addressed (MLHFQ)**
		common in patients with heart failure. (HF Assoc /ESC 2012 Tab27)			
**Anxiety**		Provide psychosocial support to patients and family and/or caregivers. HF Assoc<br><br>A major problem, esp. close to diagnosis. “Heart failure” has very frightening connotations /not knowing makes you anxious. (FG)<br><br>Breathlessness is frightening (things go round in your head) (FG)	Recognising and taking steps to manage depression, anxiety and other mental health aspects. (StQ-HPsych)		

**Programme objectives** (behl, psychl, envl outcomes to be targeted)	**Nurses**	**Patients /patient groups**	**Other**	**Guidance / evidence**	**QoL domain addressed (MLHFQ)**
**Cognitive function /memory problems**		Learn about treatment options if appropriate (HF Assoc /ESC 2012 Tab27)<br><br>Understand that cognitive dysfunction is common in patients with heart failure. (HF Assoc /ESC 2012 Tab27)			
**Psychological adjustment /change in self concept**		It's a bit of a shake up, so you need to put things back in order /get things back on track. (FG)<br><br>You can't do the things you used to do (FG)<br><br>May need to make	The aim here should be to help the individual to adapt his or her lifestyle, life goals and expectations to the challenge presented by heart failure. The individual should be able to move from being a person disrupted by heart failure to being a person living with and coping with heart failure. The sense of attaining some form of 'good-enough' control, a sense of 'safety' and acceptance of the limitations and the inherent	Include a "psychological and educational component". (CG108)<br><br>People's reactions and responses to the development of heart failure vary considerably. Explaining and helping people to move	

**Programme objectives** (behl, psychl, envl outcomes to be targeted)	**Nurses**	**Patients /patient groups**	**Other**	**Guidance / evidence**	**QoL domain addressed (MLHFQ)**
		compromises (FG)<br><br>Need to find other ways to get an enjoyable, healthy, active life (FG)<br><br>“What we have to recognise is a new norm” (FG)	uncertainty of heart failure seem to be important here. This may include finding (or re-defining) the individual’s role within the home and in the wider world and in relation to friends /family. (Qual MetaSyn, StQ-HPsych)	through the ‘five stage process’ of disruption, conception, reaction, response and assimilation might be helpful here. (Qual MetaSyn)	
**Sleeping well**		Engage in preventive behaviour such as reducing weight in obese patients, smoking cessation, and abstinence from alcohol. (HF Assoc, ESC 2012 Tab27)<br><br>I stop breathing at night /fell more breathless at		Sleep apnoea (breathing problems during sleep) should be recognised and steps taken to manage it. This may involve weight loss in severely overweight persons, smoking cessation, abstinence from alcohol, or use of CPAP treatment (breathing masks) if needed. Elevation of the head during sleep,	

**Programme objectives** (behl, psychl, envl outcomes to be targeted)	**Nurses**	**Patients /patient groups**	**Other**	**Guidance / evidence**	**QoL domain addressed (MLHFQ)**
		might /when I lie down (FG)<br><br>Learn about treatment options if appropriate. (HF Assoc, ESC 2012 Tab27)		and changing of eating and timing of diuretic medication may also be considered. (ESC,2008?)	
**Monitor symptoms /progress**	What they can do to be in control of their condition i.e monitoring weights, fluid restriction when appropriate etc. (StQ: HFN, HF/CR SN)	Monitor and recognise signs and symptoms and QoL. (HF Assoc, in ESC 2012)<br><br>Record daily weight and recognize rapid weight gain. (HF Assoc, in ESC 2012)<br><br>Patients would like to get feedback on some kind of clinical marker –	People with severe symptoms should (if recommended by HP) restrict fluid intake or take other measures to manage their fluid status. (StQ-HPsych)<br><br>Need to specify what "severe symptoms" are. Also include symptoms of dizziness/ lightheadedness? (StQ HF/CR SN)<br><br>Self-monitoring of symptoms, energy levels and other important outcomes to identify possible problems and solutions. (StQ HPsych)<br><br>Self monitoring and how to recognise early signs of decompensation of HF ie orthopnoea/paroxysmal nocturnal dsypnoea/cough/oedema/ascites/inc	Self-care of heart failure can be defined as actions aimed at maintaining physical stability, avoidance of behaviour that can worsen the condition, and detection of the early symptoms of deterioration (ESC, 2008)<br><br>In the case of increasing dyspnoea or oedema or a sudden unexpected weight gain of >2 kg in 3 days, patients may increase their diuretic dose	

**Programme objectives** (behl, psychl, envl outcomes to be targeted)	**Nurses**	**Patients /patient groups**	**Other**	**Guidance / evidence**	**QoL domain addressed (MLHFQ)**
		maybe BNP (but £25 a pop) – otherwise, can monitor progress in terms of QoL measure /how you are feeling (HF)<br><br>- maybe monitor BP, energy levels, mood, PA? (HPsych)<br><br>This is learnt as time goes on (StQ Patient)<br><br>This needs to be part of regular reviews in primary care (StQ Patient)<br><br>Recognize the common side effects of each drug prescribed (HF Assoc, ESC	SOB etc etc. And contact number so that patient can report this promptly to the Heart Failure team. (StQ: HF /CR Specialist Nurse)<br><br>Chest pain management (StQ: HF/CR SN)	and/or alert their healthcare team (HF Assoc, ESC 2012, Tab 27)	

**Programme objectives** (behl, psychl, envl outcomes to be targeted)	**Nurses**	**Patients /patient groups**	**Other**	**Guidance / evidence**	**QoL domain addressed (MLHFQ)**
		2012, Tab27)			
**People with severe symptoms should (if recommended by HP) restrict fluid intake or take other measures to manage their fluid status**			StQ approval 46% (and 38% think it should be included, but not for all patients.(Mixed sources)<br><br>NB: This should be linked to monitoring of symptoms (HPsych)		
**Manage Breathlessness**		Learn about treatment options if appropriate. HF Assoc<br><br>Learn how to assess your level of breathlessness and to be able to exercise at the right level /and to slow down before it gets out of control (FG)<br><br>Breathlessness is frightening (FG)	Approval in StQ1 was 57/29% (StQ: Mixed sources)		

**Programme objectives** (behl, psychl, envl outcomes to be targeted)	**Nurses**	**Patients /patient groups**	**Other**	**Guidance / evidence**	**QoL domain addressed (MLHFQ)**
		Need to be able to recognise symptoms leading up to exhaustion (FG)			
**Manage /organise the home and work environments**		Suddenly the house you live in might not be suitable to live in – may need to live downstairs or even move house (FG)	Managing the Work environment might also be relevant for some people (access, workload, physical requirement). (REACH expert group workshop)		
**Manage financial implications**			Information about benefits that may be available and how to claim them. (StQ 67/19% approval: Mixed sources)<br><br>Managing the financial burden (change in earnings, claiming benefits). (REACH expert group workshop)		
**Smoking**	Stop! (StQ: SNx5)	Avoid secondary smoking (StQ Patient)	StQ approval 53% (and 33% felt it should be an 'as needed' module). (StQ Mixed sources)	Stop. (CG108)	

**Programme objectives** (behl, psychl, envl outcomes to be targeted)	**Nurses**	**Patients /patient groups**	**Other**	**Guidance / evidence**	**QoL domain addressed (MLHFQ)**
			Important to keep this in as smoking may be unnecessary as some patients may be non smokers only temporarily. (HMO Expert)		
**Engage in sexual activity if desired**		Be re-assured about of the safety of engaging in sexual activity, if desired. Explore concerns about sexual activity.(HF Assoc /ESC,2012 Tab 27)	Is erectile dysfunction a problem here? Can they use meds like Viagra? Who can they discuss such topics with?<br><br>StQ1 for reassurance about engaging : approval 56 /34% (StQ: Mixed sources)	Discuss sexual activity if relevant to patient.(CG108)<br><br>Be aware of and able to use possible strategies for prevention of dyspnoea (breathing difficulties) and chest pain during sexual activity (e.g. nitroglycerine tablets).(ESC 2008?)<br><br>Women with HF should (if applicable) be aware of the potential risks associated with pregnancy and should discuss family planning	

**Programme objectives** (behl, psychl, envl outcomes to be targeted)	**Nurses**	**Patients /patient groups**	**Other**	**Guidance / evidence**	**QoL domain addressed (MLHFQ)**
				issues with a physician. (ESC,2008?)<br><br>Erectile dysfunction should be treated in the usual way; phosphodiesterase V inhibitors are not contraindicated other than in patients taking nitrates. Indeed these agents have favourable haemodynamic effects in patients with HF-REF. Phosphodiesterase V inhibitors may cause worsening LV outflow tract obstruction in patients with hypertrophic cardiomyopathy,which may be a concern in some patients with HF-PEF. (ESC 2012)	

**Programme objectives** (behl, psychl, envl outcomes to be targeted)	**Nurses**	**Patients /patient groups**	**Other**	**Guidance / evidence**	**QoL domain addressed (MLHFQ)**
**Understanding the condition and its treatment /management**	The earlier misconceptions are cleared up the better. (SCM-SN)<br>Does high altitude include air travel? Need to be clear who to discuss travel with (StQ-GP, HFN, Patient FG).	Heart failure" suggests that there is no recovery. Need to address this early (asap!). (FG)<br>Understand the cause of heart failure and why symptoms occur. (HF Assoc, in ESC 2012)<br>Knowing symptoms /what it means. What are the symptoms that should ring alarm bells for a) stroke b) heart attack c) fluid retention /decompensation d) side effects . "is it heart problems or just old age?"	The earlier misconceptions are cleared up the better. (HPsych)<br>What can be done to help and stress that HF doesn't mean they will drop dead tomorrow is what most patients want to know. (SCM)<br>Understanding what each medication is for and when to take them and also what effects or side effects to expect (StQ-HPsych)<br>Addressing concerns about medications, other treatments /interventions and their side effects. (StQ-HPsych)<br>The need for the doctor or nurse to change the dosage or type of medications over time should also be understood.(StQ-HPsych)<br>The details for air travel can quickly get out of date, so need to update facilitator training regularly. (StQ-HMO Expert)<br>Over the counter medicines to avoid	"The goals of treatment in patients with established HF are to relieve symptoms and signs (e.g. oedema), prevent hospital admission and improve survival." (ESC 2012)<br>Understand the cause of heart failure and why symptoms occur. (ESC 2012, Table 27)<br>Understand prognosis /prognostic factors and make realistic decisions (ESC 2012, Table 27)<br>Offer "education" tailored to patient needs. CG108<br>Air travel (CG108)<br>Monitor for med side effects, fluid status, functional capacity,	

**Programme objectives** (behl, psychl, envl outcomes to be targeted)	**Nurses**	**Patients /patient groups**	**Other**	**Guidance / evidence**	**QoL domain addressed (MLHFQ)**
		(FG)<br><br>Know how and when to notify healthcare provider. (HF Assoc, FGx2)<br><br>Understand indications, dosing, and effects of drugs. (HF Assoc, ESC 2012)<br><br>Know about side effects and what can be done to reduce them (FG)<br><br>When travelling, carry a written medical history and current medication regimen and extra medication. (HFAssoc, ESC	because of possible worsening of heart failure (StQ: Pharmacist)<br><br>Management of skin/oedema (StQ: HF/CR SN)<br><br>Blood tests – why and when they are important (StQ: Pharmacist)<br><br>Evidence base for medicines not as robust for HFPEF as for LVSD therefore medication advice may differ. Similarly for devices etc (StQ: HF/CR SN)<br><br>Understanding of important medical terms such as ‘congestion’, ‘decompensation’. May not be essential, but could be helpful for some. (HPsych)	cognitive status, nutrition. CG108 *(patient, nurse, carer)*<br><br>Know when /how to seek help (emergency and non-emergency). CG108<br><br>Discuss prognosis. CG108<br><br>High altitudes (1500 m or more) and travel to very hot and humid destinations should be discouraged for symptomatic patients. Planned travel should be discussed with the healthcare team. (ESC,2008?)<br><br>NSAIDs should be avoided if possible as they may cause sodium and water retention, worsening	

**Programme objectives** (behl, psychl, envl outcomes to be targeted)	**Nurses**	**Patients /patient groups**	**Other**	**Guidance / evidence**	**QoL domain addressed (MLHFQ)**
		2012 Tab27)<br><br>Prepare travel and leisure activities according to physical capacity. (HFAssoc, ESC 2012 Tab27)<br><br>Monitor and adapt fluid intake particularly in hot climates. Beware adverse reactions to sun exposure with medications (e.g. amiodarone). HF Assoc<br><br>Sometimes need specialist advice to help with medication dosing /adjustment (FG)<br><br>What is heart		renal function and worsening HF (ESC 2012)<br><br>"The aim of using diuretics is to achieve and maintain euvolaemia (the patient's 'dry weight') with the lowest achievable dose." (ESC 2012)<br><br>"This means that the dose must be adjusted, particularly after restoration of dry body weight, to avoid the risk of dehydration leading to hypotension and renal dysfunction" (ESC 2012)	

**Programme objectives** (behl, psychl, envl outcomes to be targeted)	**Nurses**	**Patients /patient groups**	**Other**	**Guidance / evidence**	**QoL domain addressed (MLHFQ)**
		failure (need this info at diagnosis). How does it occur? What are the symptoms? Why do some people get heart failure and others not? (FG)<br><br>What is the problem and what can I do about it? (FG)<br><br>Put things in layman's terms /non-technical language (FG)<br><br>How much better can I expect to get from where I am now? (FG)<br><br>Knowing how to explain the problem			

**Programme objectives** (behl, psychl, envl outcomes to be targeted)	**Nurses**	**Patients /patient groups**	**Other**	**Guidance / evidence**	**QoL domain addressed (MLHFQ)**
		/symptoms to HPs (FG)<br><br>Need to understand why the fluids build up and what to do about it. (FG)<br><br>We're all different – some hearts beat too slow, some too fast – some need implants, some don't – So information needs to be tailored. (FG)<br><br>What side effects might medications cause (e.g. panic attacks) – can they be reduced (e.g. by changing medication). When to contact			

**Programme objectives** (behl, psychl, envl outcomes to be targeted)	**Nurses**	**Patients /patient groups**	**Other**	**Guidance / evidence**	**QoL domain addressed (MLHFQ)**
		HP about side effects. (FG)			
**Diet and weight management**		Sodium restriction may help control the symptoms and signs of congestion in patients with symptomatic heart failure classes III and IV. HF Assoc<br><br>Eat healthily and keep a healthy weight. HF Assoc<br><br>Monitor and prevent malnutrition. HF Assoc<br><br>Avoid excessive fluid intake in severe HF. HF Assoc	Make changes to diet to reduce weight if Body Mass Index is more than 30Kg/m2. However, this is not recommended for people with evidence of cachexia (involuntary weight loss /body wasting).(StQ1)<br><br>76% (and 14%) approval for eating healthy and maintaining a healthy body weight (StQ mixed sources)	Some approaches may not be beneficial, e.g. advice to restrict sodium intake. ESC 2012<br><br>Obesity should be managed as recommended in other guidelines (ESC 2012)<br><br>Iron deficiency may contribute to muscle dysfunction in HF and causes anaemia. May be treated with iron therapy (supplements) (ESC 2012)	
**Alcohol**		Modest intake of		Patients with alcohol-	

**Programme objectives** (behl, psychl, envl outcomes to be targeted)	**Nurses**	**Patients /patient groups**	**Other**	**Guidance / evidence**	**QoL domain addressed (MLHFQ)**
		alcohol (2 units per day in men or 1 unit per day in women): abstinence for patients with alcohol-induced cardiomyopathy. HF Assoc		related heart failure to abstain. CG108<br><br>Discuss consumption with other patients and tailor advice to circumstances. CG108	
**Taking meds**		Increase diuretic dose and/or alert healthcare team if increasing dyspnoea or oedema or sudden unexpected weight gain (>2 kg in 3 days). (HF Assoc, in ESC 2012, Tab 27)<br><br>Use flexible diuretic therapy if appropriate and recommended		Titration of diuretics, **as needed**, for congestion and fluid retention. CG108<br><br>Regular taking of other meds, as outlined in CG108<br><br>Many patients can be trained to self-adjust their diuretic dose, based on monitoring of symptoms/signs of congestion and daily weight measurements. (ESC 2012)	

**Programme objectives** (behl, psychl, envl outcomes to be targeted)	**Nurses**	**Patients /patient groups**	**Other**	**Guidance / evidence**	**QoL domain addressed (MLHFQ)**
		after appropriate education and provision of detailed instructions. (HF Assoc, ESC 2012, Tab 27)<br><br>The aim is to use medications in the best way to help you function positively (StQ)<br><br>Useful to talk with the pharmacist. Discuss changes, ask for bubble packs (pre-packed daily medication combinations) (HF)<br><br>Understanding of dose changes and titration is too much but a 'how		Patient involvement in symptom monitoring and flexible diuretic use (HFA and Table 26 in ESC 2012)<br><br>In the case of increasing dyspnoea or oedema or a sudden unexpected weight gain of >2 kg in 3 days, patients may increase their diuretic dose and/or alert their healthcare team (HF Assoc, ESC 2012, Tab 27)	

**Programme objectives** (behl, psychl, envl outcomes to be targeted)	**Nurses**	**Patients /patient groups**	**Other**	**Guidance / evidence**	**QoL domain addressed (MLHFQ)**
		to' approach to identify when things need to change could work.(StQ: SN)<br>Self-titration should be optional for those patients who feel they would like to take this on. (StQ: SCM)<br>Some patients may not be comfortable adjusting dose themselves, so need to agree a strategy with the HFN (FG)			
**Correct use of devices (e.g. Implantable Cardiac Defibrillator,**			Understanding how to manage and respond to device alarms, where appropriate. StQ Approval 14 /71%,so recommended where such devices are in use. (Stq: Mixed		

**Programme objectives** (behl, psychl, envl outcomes to be targeted)	**Nurses**	**Patients /patient groups**	**Other**	**Guidance / evidence**	**QoL domain addressed (MLHFQ)**
**Cardiac Resynchronization Therapy).**			sources)<br>The nurse's role here may be to simply identify the problems and then refer for more specialist advice. (HPsych)		
**Communication with Health Professionals**			How to achieve clear and appropriate communication with healthcare professionals (StQ HPsych)		
**Helpseeking**	We need a big push on early warning signs (StQ: HF/CR SN)	When to contact HP about side effects. (FG)<br>This is learnt as time goes on (StQ-Patient) –<br>This needs to be part of regular reviews in primary care. (StQ-Patient)	Ask for help from (your heart failure nurse, GP) if you are not coping (StQ)<br>**You should seek help from a GP or HF nurse when you:-**<br>- Have sudden Weight gain that does not respond to adjustment of diuretic dosage (CRN)<br>- Feel the symptoms or worries are hindering your confidence to effectively self-manage.<br>- Have an increase in breathlessness	Deterioration in symptoms indicates heightened risk of hospitalization and death, and is an indication to seek prompt medical attention and treatment. (ESC 2012)<br>Know how and when to notify healthcare provider (HF Assoc, ESC 2012, Tab 27)	

**Programme objectives** (behl, psychl, envl outcomes to be targeted)	**Nurses**	**Patients /patient groups**	**Other**	**Guidance / evidence**	**QoL domain addressed (MLHFQ)**
			or fatigue, especially when at rest<br><br>- Feel that the plan isn't working and QoL is deteriorating<br><br>- Consistently feel ''down or in a low - mood''<br><br>- Notice a step change in your ability to do basic daily activities<br><br>- Are unable to take medication – need to say for what reasons (Patient FG)<br><br>- Are on Warfarin and have bruising or bleeding (Patient)<br><br>- or your partner /caregiver have further questions and concerns about the condition or about planned investigations /procedures. (HFN)<br><br>- Have serious problems sleeping or breathing during sleep (CG)<br><br>- Your partner/carer is struggling to cope or has medical needs of their own that are worsening.(Pharmacist)<br><br>- Experience episodes of vomiting		

**Programme objectives** (behl, psychl, envl outcomes to be targeted)	**Nurses**	**Patients /patient groups**	**Other**	**Guidance / evidence**	**QoL domain addressed (MLHFQ)**
			and diarrhoea (they may need adjustment of diuretic dosages). (HF Nurse)<br><br>- Have worsening shortness of breath /PND or oedema (HF/CR SN)<br><br>- Have increasing frequency of chest pain (HF/CR SN)<br><br>- Have an increase in sputum expectoration/ dirty sputum (HF/CR SN)<br><br>- Have unusual palpitations (HF/CR SN)<br><br>- Experience symptoms which might indicate side effects of drug therapy (Pharmacist)<br><br>(StQ, Mixed sources)<br><br>- A guidance booklet or card (optional) and a page in the manual formatted as a 'traffic light' system may be appropriate here (HPsych)<br><br>- Traffic light system is a definite		

**Programme objectives** (behl, psychl, envl outcomes to be targeted)	**Nurses**	**Patients /patient groups**	**Other**	**Guidance / evidence**	**QoL domain addressed (MLHFQ)**
			must as most of us are now using this approach and resource now available via CHSS. (HF/CR SN)<br><br>**You should seek emergency care (go to hospital or call an ambulance) if you have:-**<br>- Loss of consciousness<br>- Chest pains or sudden onset of palpitations (potential arrhythmia)<br>- If ICD (implanted defibrillator) shocks are applied (for patients with these devices)<br>- Unrelieved shortness of breath while sitting still (HFN x 2)<br>- Acute increase in shortness of breath (HFN)<br>- Coughing up frothy, pink sputum (HFN)<br>- Distressing sudden breathlessness especially at night (HF/CR SN)<br>- Patient not responding to treatment (HF/CR SN)		

**Programme objectives** (behl, psychl, envl outcomes to be targeted)	**Nurses**	**Patients /patient groups**	**Other**	**Guidance / evidence**	**QoL domain addressed (MLHFQ)**
			A guidance page or (optional) booklet formatted as a 'traffic light' system may be appropriate here (H Psych)<br>Ensure DNR discussed or covered somewhere in booklet (HF/CR SN)<br>I'd divide emergency care into a) blue light and b) GP or out of hours GP callout. For example, anuria may not be a blue light problem, but the GP would certainly want to know within a few hours. (Pharmacist)		
**Engaging support from others (e.g. friends, family) to support self-care**	Need to be sure not to undermine confidence for people who don't have support.	Need to be careful not to push this as being essential. Need re-assuring messages /alternatives for people with no social support" (1. Patient FG 2. SCM)<br>Access to support from others in the	This should include ideas about information support, emotional support and practical support.(StQ-HPsych)<br>Engaging support: This can be done in the manual. A separate resource has been popular (patient audit feedback). (StQ-HMO Expert)<br>Negotiation of the role of any caregivers /supporters. (StQ 61/19%: Mixed sources)		

**Programme objectives** (behl, psychl, envl outcomes to be targeted)	**Nurses**	**Patients /patient groups**	**Other**	**Guidance / evidence**	**QoL domain addressed (MLHFQ)**
		same situation should be facilitated. E.g. through access to a local support group. (Patient FG)			
**Maintain social roles /social relationships**		Maintain social roles /relationships. (Interviews with caregivers)	Maintain social roles /relationships. (REACH expert group workshop)		
**Managing co-morbidities (other illnesses) that might affect the ability to manage heart failure**			Approval in StQ1 was 38/47%. (StQ Mixed sources)<br>Over one third of CVD patients have one or more significant co-morbidities including COPD and arthritis. Co-morbidity is linked with poor prognosis and poor recruitment to lifestyle initiatives (SCM).<br>This may include strategies for remembering to take medications regularly (e.g. pre-loaded pill boxes) and discussion of options with a	Important co-morbidities include anaemia, angina, arthritis, diabetes, COPD and asthma, cachexia, cancer, depression, erectile dysfunction, gout, hypertension, hyperlipidemia, iron deficiency, kidney dysfunction, renal dysfunction, sleep	

**Programme objectives** (behl, psychl, envl outcomes to be targeted)	**Nurses**	**Patients /patient groups**	**Other**	**Guidance / evidence**	**QoL domain addressed (MLHFQ)**
			pharmacist. (HPsych)<br><br>Mobility limitations (from arthritis or otherwise) are also important because they can interfere with /act as barriers to exercise-based rehabilitation. (HPsych)	disturbance, prostatic obstruction and obesity. (ESC?)<br><br>Management of co-morbidities is a key component of the holistic care of patients with HF (ESC 2012)<br><br>Co-morbidities may affect the use of HF treatments; drugs used to treat co-morbidities may cause worsening of HF or interact with HF drugs (e.g. NSAIDS); and they may worsen HF outcomes (ESC 2012)<br><br>Obesity should be managed as recommended in other guidelines (ESC 2012)	
**Manage end of life issues**	It is worth discussing	Prognosis – how long have I got?	Even if we decide to exclude end stage HF patients there may be	Ensure opportunity at all stages of care to	

**Programme objectives** (behl, psychl, envl outcomes to be targeted)	**Nurses**	**Patients /patient groups**	**Other**	**Guidance / evidence**	**QoL domain addressed (MLHFQ)**
	mortality and the possibility of sudden death. Carers in particular are disappointed /shocked that they were not prepared for this when it happens and they haven't had time to engage in any end of life planning. (CRN)	(FG)	concerns about the palliative stage that people may have even if they are at an earlier stage in their illness which should be addressed either in the manual or via the facilitator e.g. decisions about deactivating ICD, do not resuscitate (DNR) orders and living wills. (H Psych)	discuss issues of sudden death and living with uncertainty. CG108<br><br>Identify and manage palliative care needs as soon as possible. CG108<br><br>Ensure access to HPs with skills in palliative care. CG108<br><br>Frequent assessment of patient's physical, psychological, and spiritual needs. ESC2012<br><br>Focus on complete symptom relief from both HF and other co-morbidities. ESC2012<br><br>Advanced care planning, taking account of preferences for place of death and	

**Programme objectives** (behl, psychl, envl outcomes to be targeted)	**Nurses**	**Patients /patient groups**	**Other**	**Guidance / evidence**	**QoL domain addressed (MLHFQ)**
				resuscitation (which may include deactivating ICD). ESC2012<br><br>The nurses' role here may be simply to identify and refer.	

Other cross-cutting ideas (and source):

NB: The following may need to be incorporated into Table 1 as Programme Outcomes /Objectives, or they may pertain to the content or delivery of the training manual /training course rather than the HF Manual itself

If the patient agrees, families and carers should have the opportunity to be involved in decisions about treatment and care. (NICE CG108)

Treatment and care should be patient-centred, taking into account patients' individual needs and preferences. (NICE CG108)

Treatment should be delivered as part of an integrated approach to care delivered by a multidisciplinary team. (NICE CG108)

A meta-analysis of RCTs suggests that structured telephone support in addition to conventional care may reduce the risk of hospitalization in patients with HF. (ESC guidance, 2012)

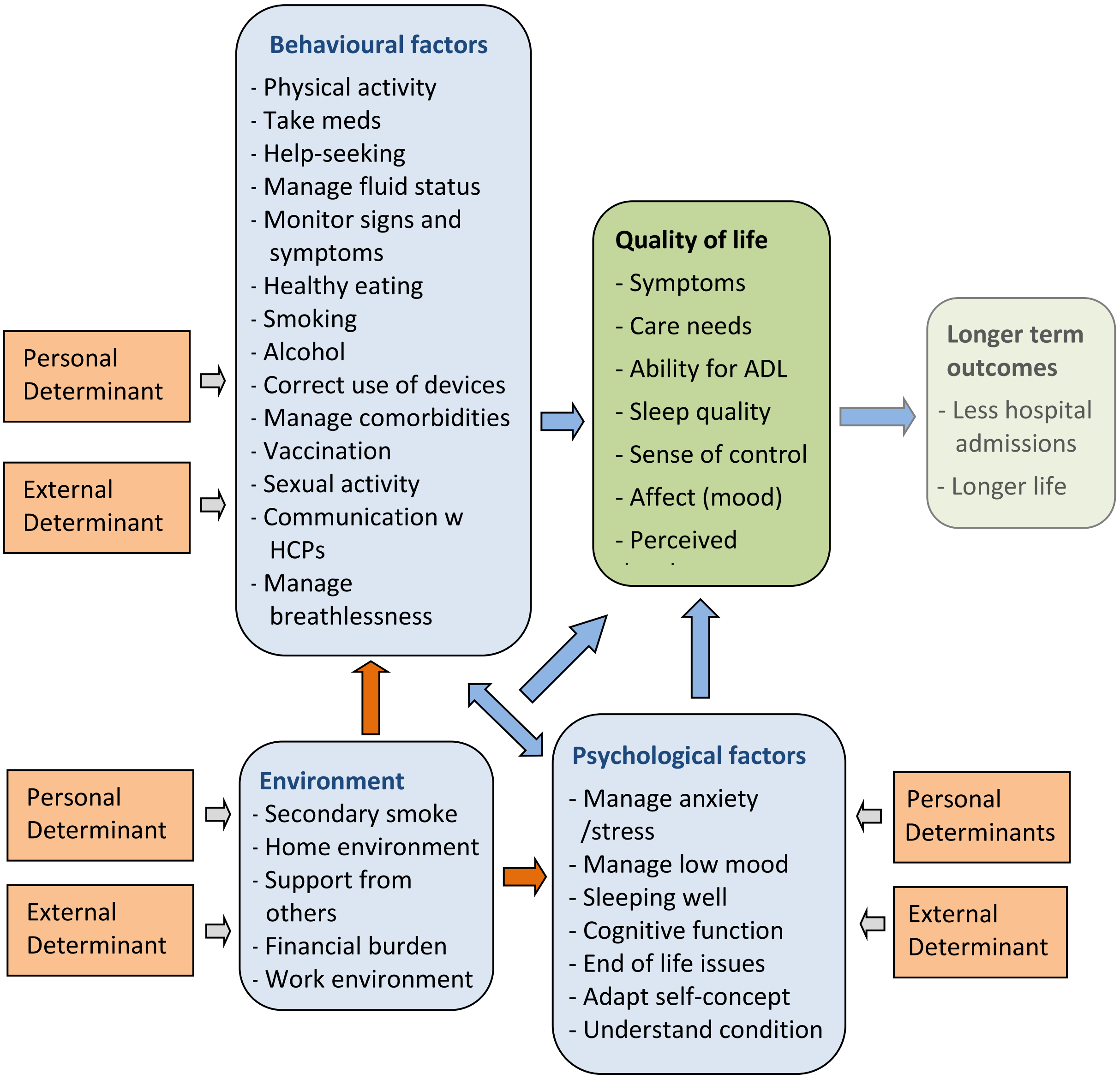

Fig. 2: Applying the PRECEDE planning model to the self-management of heart failure:
The following diagram shows how the programme outcomes /objectives identified above relate to the overall intervention model

Over-arching themes:

1. Engaging with the intervention

2 .Ongoing (lifelong) adherence to self-care behaviours
