# Supplementary material for: Optimising self-care support for people with heart failure and their caregivers: development of the Rehabilitation Enablement in Chronic Heart Failure (REACH-HF) intervention using intervention mapping
Source: Pilot Feasibility Stud. 2016 Aug 2;2:37. doi: 10.1186/s40814-016-0075-x (PMC5153822; doi:10.1186/s40814-016-0075-x)
Supplement: Additional file 3: — Extracts from intervention maps for stress management, medication management and managing symptoms. (PDF 267 kb) [file 40814_2016_75_MOESM3_ESM.pdf]

Additional File 3: Extracts from intervention maps for stress management, medication management and managing symptoms (NB: These are only selections from the full intervention maps for each topic).

Table 3.1 Extract from the REACH-HF intervention map for the change target "Support patients and /or caregivers to manage stress, anxiety and breathlessness". NB: this is only a selection from the full intervention map for this change target. Other performance objectives (not shown) were "3. Ongoing use (maintenance) of stress recognition and stress management actions; 4. Prevention of stress; 5. Recognise when breathlessness (due to acute stress or anxiety) becomes problematic; 6. Manage breathlessness".

Performance Objective	Modifiable determinants of change	Change Techniques	Strategies
1. Recognise problematic levels of stress	*Knowledge /motivation:*- Belief that it is worth bothering about (perceived	[b] Provide information on identity, timeline, causes, consequences and control to build a functional	Nurse to check for and address misconceptions. What do you know about how stress affects your heart condition? (F)

**Performance Objective**	**Modifiable determinants of change**	**Change Techniques**	**Strategies**
(including acute and chronic stress and anxiety)<br><br>*For action by:*<br>Patient<br>Facilitator<br>Caregiver	importance and treatment efficacy)<br><br>Knowledge of signs and symptoms and different types of stress (chronic, acute)<br><br>Understanding what is normal and what might be problematic	understanding /illness model (how stress works and how to manage it)<br><br>[a] Provide information on consequences<br><br>[b] Simultaneous self-monitoring of behaviour and symptoms (linking use of stress-management techniques to stress levels, sleep quality and HF symptoms; linking physical activity and stress	Explain how stress affects heart failure symptoms (and vice-versa). Explain how stress works and how it can be managed (give hope!) (F,M)<br><br>Provide normative information about others' experiences of stress. Can include a good news case study or Patient Voices (quotes) "having a heart problem made me realise what was important in life – I don't fret about the small stuff so much these days" (M)

Performance Objective	Modifiable determinants of change	Change Techniques	Strategies
		levels)	Provide information on behavioural patterns indicative of stress (e.g. via case study) (M)
	Skills:<br>Self-awareness (skills for symptom recognition /categorisation)<br><br>Prior experience of recognising and managing stress	[a] Model/ demonstrate the behaviour (through case study)<br><br>[a] Set graded tasks (gradual capacity building for stress-recognition /categorisation skills)<br><br>[a] Prompt practice<br><br>[b] Self-monitoring of symptoms	Self-assessment using HADS or stress assessment scale (M)<br><br>Facilitated assessment if needed using HADS or stress assessment scale (F)<br><br>Self-assessment of stressors (what causes stress for the individual /what the most common causes are – e.g. sleep disturbance, palpitations; mood swings; concentration

Performance Objective	Modifiable determinants of change	Change Techniques	Strategies
			issues; lack of coping strategies) (M)<br><br>Nurse to Identify existing strengths /skills and positive attitudes (F)
	Social /contextual:<br>Social support /encouragement	[a] Plan social support<br><br>[a] Prompt barrier identification and problem solving<br><br>[b] Engage health care support	Problem-solving in relation to engaging social support /seeking help if needed (F)<br><br>Encourage P to open up a conversation with significant other and vice versa (discuss how patient may be coping/how carer may be coping) (F, M, CGM)

**Performance Objective**	**Modifiable determinants of change**	**Change Techniques**	**Strategies**
	Other:<br>Avoidance of the issue (e.g. due to fear of consequences)<br><br>Side effects of medication (affecting cognitive function, or as a direct cause of anxiety /stress symptoms)	[b] Cognitive behaviour therapy (to address reasons for denial /minimisation)<br>[b] Referral for medical intervention	Briefly reflect on /explore reasons for avoidance of the issue (need to be careful not to exceed professional competencies here). Link to manual sections on living with uncertainty /end of life issues) (F)<br><br>Consider medications as a possible factor and prompt to seek advice on changing meds if appropriate (F)<br><br>Signpost to contact with other health professionals /other services if needed (F)

Performance Objective	Modifiable determinants of change	Change Techniques	Strategies
2. Manage stress (including acute and chronic stress and anxiety).<br><br>i.e. Taking action once abnormal stress has been identified<br><br>*For action by:*	*Knowledge /motivation:*<br>- Perceived importance (why should stress be managed?)<br>- Treatment efficacy (belief that something can be done about it)<br>- Knowledge about how to access appropriate help<br>- Self-efficacy (can I do it? (e.g. relaxation techniques))	[b] Provide information on identity, timeline, causes, consequences and control to build a functional understanding /illness model (how stress works and how to manage it)<br><br>[a] Provide information on behaviour /health link<br><br>[a] Provide information about consequences of behaviour	See section above (*PO1 Knowledge*): Nurse to check for and address misconceptions (F); Explain how stress affects heart failure symptoms, how stress works and how it can be managed (F,M); Provide normative information about others' experiences of stress (M); Provide information on behavioural patterns indicative of stress (M);<br><br>Outline evolutionary purpose of stress and illustrate how that can be maladaptive today. (e.g. fight or flight reaction) (M)

**Performance Objective**	**Modifiable determinants of change**	**Change Techniques**	**Strategies**
Patient<br><br>Caregiver		[a] Prompt intention formation (for stress management)	Reinforcement from facilitator (and in relaxation CD) promoting the importance of stress management (F, M)<br><br>Prompt self-monitoring of outcomes and relate progress /changes to actions (to help understand what works /what doesn't work for the individual) (M, F)<br><br>F to discuss /summarise reasons for engaging in stress-management and prompt intentions for stress management /following text in the

Performance Objective	Modifiable determinants of change	Change Techniques	Strategies
			manual on potential benefits (M, F)
	Skills<br><br>Existing stress management skills and prior experience	[a] Stress management (multiple techniques)<br><br>[a] Provide instruction (how to do relaxation techniques)<br><br>[a] Prompt practice (of stress management skills /relaxation techniques)	Text in manuals and CD with selected, easy to learn /use, mindfulness and techniques and other alternatives (e.g. progressive relaxation, diaphragmatic breathing, visualisation) for managing acute stress (M)<br><br>Prioritisation of what is really important to achieve in a day/week (M)<br><br>Encourage communication with others to resolve sources of conflict (e.g. using

**Performance Objective**	**Modifiable determinants of change**	**Change Techniques**	**Strategies**
		[a] Prompt practice (of pleasurable activities)<br><br>[a] Set graded tasks<br><br>[a] Prompt specific goal setting<br><br>[b] Prompt self-monitoring of outcomes<br><br>[a] Prompt barrier identification and	assertiveness methods to outline personal needs and negotiate support). Esp. discussion between cared for and caregiver (M, F, CGM))<br><br>Provide information about when to seek help from GP and counselling or mental health services (M, F)<br><br>Provide checklist of ‘red flag’ signs to prompt appropriate help-seeking (M, F)

Performance Objective	Modifiable determinants of change	Change Techniques	Strategies
		problem solving<br><br>[a] Model/Demonstrate behaviour<br><br>[a] Teach to use prompts/cues<br><br>[a] Relapse prevention	Plan for 'Me' time – within the home and outside the home (M, CGM)<br><br>Tips on avoiding stressors – e.g. Where available /relevant, sleeping in a separate room may allow better sleep; listening to music, reading, spending time with family/ pets, hobbies: gardening etc (M, F)<br><br>Nurse support during the intervention and referral to an alternative support system (if needed) at the end of the intervention (F)

**Performance Objective**	**Modifiable determinants of change**	**Change Techniques**	**Strategies**
			Demonstrate selected relaxation /stress management techniques including breathing & progressive relaxation if useful (F)<br><br>Encourage social Interaction /having fun as a way to ameliorate stress (M)<br><br>Prompt identification of opportunities for practising stress management by giving examples via case studies or tips (M, F)

Performance Objective	Modifiable determinants of change	Change Techniques	Strategies
			Address & support solutions to barriers /resistance to practising stress management via discussion (F) and providing examples via case studies or tips (M)<br><br>Prompt use of environmental cues to remind individuals to carry out stress management activities. Provide tools to practice stress reduction e.g. relaxation CD with exercises to follow (M, F)
	Social /contextual:	[a] Plan social support	Promote use of social support structures –

Performance Objective	Modifiable determinants of change	Change Techniques	Strategies
	- Social support /encouragement<br>- Lack of time (for practice)<br>- Access to referral (if needed) to appropriate health service	[b] Engage healthcare support	texting, support groups, extended family, friendships (M, F, CGM)<br><br>‘Patient Voices’ in quotes /illustrations on the importance of managing stress and benefits of using relaxation techniques.<br><br>Signpost to sources of respite care to help with caregiver stress if needed (F, CGM)<br><br>If needed, Nurse to facilitate referral to appropriate health service provider (e.g. for

**Performance Objective**	**Modifiable determinants of change**	**Change Techniques**	**Strategies**
			clinical levels of depression or anxiety) (F)

*M = Manual content; F = Facilitator task; CGM = Caregiver Manual

[a] Techniques listed in the Abraham & Michie (2008) taxonomy.[1]

[b] Techniques not listed in the Abraham & Michie (2008) taxonomy.

Table 3.2 Extract from the REACH-HF intervention map for the change target "The patient (and caregiver if appropriate) should manage their heart failure (and other) medications using them in such a way as to optimise their physical condition and mental well-being /quality of life". NB: this is only a selection from the full intervention map for this change target. Other performance objectives (not shown) were "1. The patient (and any caregivers /supporters) should have a clear understanding of how to use their medication as a key part of the management of their HF; 3. Recognising and monitoring side-effects; 4. Taking appropriate action if medication missed; 5. Taking appropriate action during other illness (e.g. gastric upset, cold); 7. Self-management /self-titration of diuretics where the patient is willing and the GP /heart failure team is in agreement.

**Performance Objective**	**Modifiable determinants of change**	**Change Techniques**	**Strategies**
1. The patient should take all their medications as prescribed (and	*Knowledge /motivation:*<br>- Knowledge about what to take, how and when<br>- Beliefs about medication	[a] Provide information on consequences<br>[a] Provide instruction	Nurse (and text in manual) to explain about medication, how /why medication may change, type of medication and dose of medication (F, M)

Performance Objective	Modifiable determinants of change	Change Techniques	Strategies
consult a health professional if there are any problems in doing this)<br><br>*For action by:*<br>Patient<br>Caregiver	effectiveness (treatment efficacy /expectations of benefit or harm)<br>- Beliefs about seeking health care support if any problems<br>- Patient concerns /past experience (esp. about side effects)<br>- Carer's concerns/past experience<br>- Trust in prescriber<br>- Confusion over branding	[b] Simultaneous self-monitoring of behaviour and symptoms (linking use of medication to well-being and HF symptoms).<br><br>[a] Motivational interviewing<br><br>[b] Engage healthcare support	Provide normative information about how other people benefit /what to expect from your medication (M)<br><br>Possible case study or use of 'Patient Voices' in quotes /illustrations on attitudes to medication-taking (M)<br><br>Nurse to identify any patient concerns, problems or reasons for non-adherence, address any misconceptions and work with patient (and wider health care team if needed) to seek solutions (F)

Performance Objective	Modifiable determinants of change	Change Techniques	Strategies
	/packaging /how and how many to take<br>- Consistency of messages from different care staff (mixed messages)		Advise patient to use regular pharmacy for repeat prescriptions to minimise confusion (pharmacists don't always use the same brand) (F, M)<br><br>See sections elsewhere on making an 'emergency plan' in case of serious side effects decompensation or fluid build-up (M, F)<br><br>Travel advice: When travelling, carry a written medical history and current medication regimen and extra medication. High altitudes

Performance Objective	Modifiable determinants of change	Change Techniques	Strategies
			(1500 m or more) and travel to very hot and humid destinations should be discouraged for symptomatic patients. Planned travel should be discussed with the healthcare team. Beware adverse reactions to sun exposure with some medications. Details for air travel advice can change, so facilitator needs to stay updated (M, F)<br><br>Follow up performance (symptom changes in relation to adherence, any side effects) (F)<br><br>Encourage patient /caregiver to discuss with

Performance Objective	Modifiable determinants of change	Change Techniques	Strategies
			facilitator or GP if they have any concerns or queries about their medications, or any problems in taking them (M, F)<br><br>The nurse facilitator should check if any of the information covered conflicts with anything else the patient has been told and seek to resolve any conflicts (contacting other HPs involved if necessary) (F)<br><br>Text in manual on common barriers to taking medication and possible solutions. (M)

Performance Objective	Modifiable determinants of change	Change Techniques	Strategies
			Facilitation to elicit any other individual barriers and possible solutions(F)
	Skills<br><br>- Memory/ cognitive function	[a] teach to use prompts/cues<br>[a] Use of follow up prompts<br>[a] Prompt barrier identification (and problem-solving)<br>[a] Prompt self-monitoring of behaviour	Discuss use of dosette box or pharmacy system to help organise meds (F, M)<br><br>Include a simple check on medication adherence (and a medication plan) in the planning /monitoring resource (M)<br><br>Record changes to medication – when, who, why (M)<br><br>Facilitator to assess whether patient has

Performance Objective	Modifiable determinants of change	Change Techniques	Strategies
			cognitive abilities to take medication as prescribed (F)<br><br>Encourage use of aide-memoires for taking medication – organisation system – Diary? Alarm on phone (M, F)<br><br>Text in manual on common barriers to taking medication and possible solutions. (M)<br><br>Facilitation to elicit any other individual barriers and possible solutions(F)
	Social /contextual:	[a] Plan social support	Involve carer in monitoring medication use –

Performance Objective	Modifiable determinants of change	Change Techniques	Strategies
	- Changes in circumstances<br>- Eyesight<br>- Environment (e.g. holidays /travel)<br>- Social support (esp. caregiver and other family<br>- Presence of side-effects<br>- Support from GP /care team around managing any problems	[b] Engage health care support<br>[a] Prompt barrier identification	may need to facilitate some negotiation between caregiver and patient about what the caregiver's role is here (F, CGM, M)<br><br>See above re text on travel advice (M)<br><br>Nurse to work closely with specialist nurse, clarification of roles, clear communication strategy and pathway, facilitator to contact specialist nurse or GP re changes needed to medications /managing of side effects etc (F)<br><br>Nurse to identify any other patient concerns,

Performance Objective	Modifiable determinants of change	Change Techniques	Strategies
			problems or reasons for non-adherence, address any misconceptions and work with patient (and wider health care team if needed) to seek solutions (F)<br><br>Assess whether eyesight good enough to read labels (F)
6. Avoiding over-the-counter (OTC) medications and supplements if	• Knowledge<br>• Social support<br>• Health care support (pharmacist)	[a] Provide information on consequences (and general information)<br><br>[a] Plan social support	Prompt P and CG to ask pharmacist about *any* OTC medication or supplement purchases (M, F)<br><br>Recommend using a regular pharmacist who knows you have HF (M)

**Performance Objective**	**Modifiable determinants of change**	**Change Techniques**	**Strategies**
harmful<br><br>*For action by:*<br>Patient<br>Caregiver		[b] Engage healthcare support	Carry prescription or medication record with you to show pharmacist if unable to use the regular system (M)<br><br>Highlight that OTCs can contain a variety of medications that can affect the way your HF medications work e.g. cold remedies –Aspirin (M)<br><br>Positive messages around safe options (e.g. Paracetamol and drinking honey/lemon/water as a lay remedy for colds that soothes sore

**Performance Objective**	**Modifiable determinants of change**	**Change Techniques**	**Strategies**
			throats and is not harmful) (M)<br><br>Engage caregiver in checking /monitoring here (and also to know that they should not buy these products for the patient) (CGM)

*M = Manual content; F = Facilitator task; CGM = Caregiver Manual

[a] Techniques listed in the Abraham & Michie (2008) taxonomy.[1]

[b] Techniques not listed in the Abraham & Michie (2008) taxonomy.

Table 3.3 Extract from the REACH-HF intervention map for the change target "The patient should monitor and respond to Signs and Symptoms of Heart Failure – this is important to inform appropriate help-seeking and also for managing fluid status". NB: this is only a selection from the full intervention map for this change target. Other performance objectives (not shown) were "1. Monitor symptoms of heart failure (general); 4. Detecting other signs and symptoms (e.g. heart attacks, change in mood)".

**Performance Objective**	**Modifiable determinants of change**	**Change Techniques**	**Strategies**
2. Self-Monitor specific symptoms related to decompensation<br><br>*For action by:*<br>Patient	*Knowledge /Motivation*<br>Knowledge of signs and symptoms of decompensation<br><br>Knowledge of potential causes of decompensation<br><br>Knowledge of normal	[b] Provide information on identity, timeline, causes, consequences and control to build a functional understanding /illness model (how fluid build-up can happen in HF, the consequences and how to manage it)<br><br>[a] Provide information on	Provide a clear rationale for monitoring fluids /symptoms relating to decompensation (M, F)<br><br>Provide information on decompensation symptoms: Oedema, sudden weight gain, breathlessness, coughing, nocturnal dyspnea, fatigue etc. (M)<br><br>Provide Information on when /how to contact

**Performance Objective**	**Modifiable determinants of change**	**Change Techniques**	**Strategies**
Caregiver	/acceptable levels of symptom severity /what levels should trigger action<br><br>Belief that symptom monitoring will improve QoL (treatment efficacy)<br><br>Knowledge of potential cause of sleep disturbance in relation to symptoms<br><br>Knowledge of how and when	consequences<br><br>[a] Motivational interviewing	healthcare provider (M, F).<br><br>Quiz /checklist to help actively process information (M)

Performance Objective	Modifiable determinants of change	Change Techniques	Strategies
	to notify healthcare provider		
	Skills<br>Skills in measurement /assessment<br><br>Belief that patient can take the actions needed (self-efficacy)	[b] Prompt self-monitoring of outcomes (decompensation-specific symptoms)<br><br>[a] Barrier identification (and problem-solving)<br><br>[a] Modelling<br><br>[a] Motivational interviewing	Identify any possible barriers to self-monitoring (e.g. ask 'what might stop you'; present several typical barriers) and discuss possible solutions (NB: it is ideal if patient generates the ideas, but can also put some examples in the manual) (M, F)<br><br>Use of monitoring tools in a symptom diary or checklist. Include traffic light tool mentioned elsewhere (appropriate help-seeking section) (M). Including ..

Performance Objective	Modifiable determinants of change	Change Techniques	Strategies
			- Ankle circumference<br>- Weight<br>- Other symptoms<br><br>Case-study including daily self-monitoring (M)
	Social /contextual:<br><br>Social support (esp. people who live with patient and can help with monitoring)<br><br>Cognitive functioning	[a] Plan social support<br><br>[a] Barrier identification (and problem-solving)<br><br>[a] Motivational interviewing	Identify social and contextual /other barriers to self-monitoring and discuss possible solutions (NB: it is ideal if patient generates the ideas, but can also put some examples in the manual) (M, F)
	Other	CBT to address avoidance issue	Nurse to assess and address any reluctance

Performance Objective	Modifiable determinants of change	Change Techniques	Strategies
	Avoidance of the problem. This may stem from not wanting to think about HF due to anxiety /fear, or it may stem from not wanting to appear weak or vulnerable (self concept issues).		to engage /avoidance issues (F)<br><br>Engage caregiver /other co-habitees in helping to monitor (F, CGM)
3. .Prevention of decompensation through management of	Knowledge about decompensation and its causes (as above)	[b] Provide information on identity, timeline, causes, consequences and control to build a functional understanding /illness model	Information on what decompensation is /its causes (M, F), including<br>• Fluid overload<br>• Poor medication concordance

**Performance Objective**	**Modifiable determinants of change**	**Change Techniques**	**Strategies**
fluid status and taking medications	Self-efficacy(confidence about being able to take the actions needed)<br><br>Social support<br><br>Quality of health care support	(how fluid build-up can happen in HF, the consequences and how to manage it)<br><br>[a] Prompt specific goal setting<br><br>[a] Barrier identification /problem-solving<br><br>[a] Plan social support<br><br>Engage health care support	• Illness; infection, event e.g. MI<br><br>Information on signs and symptoms of decompensation and appropriate action to take if it occurs (or starts to build up) (M, F)<br>• Case-study<br>• Quiz<br>• Danger signs /red flag symptoms<br><br>Include a ‘traffic light’ action-planning guide as part of the self-monitoring tool to guide appropriate action – green = no problem, keep going; amber = check self-care

Performance Objective	Modifiable determinants of change	Change Techniques	Strategies
			behaviours and monitor more closely, adjust diuretic dose as instructed (or call GP or HF nurse for guidance) (maybe take it easy /manage fatigue); red = seek help urgently)<br><br>Strategies to support taking medication are specified in the "Taking medications" section.<br><br>Engage caregivers /co-habitees in fluid monitoring /management (CGM, F)<br><br>Encourage wider advice-seeking and /or help-seeking, as appropriate from health care

Performance Objective	Modifiable determinants of change	Change Techniques	Strategies
			system (F, M)<br><br>Nurse to provide emergency (and non-emergency) help numbers for using the local system and record in manual (F, M)

*M = Manual content; F = Facilitator task; CGM = Caregiver Manual

[a] Techniques listed in the Abraham & Michie (2008) taxonomy.[1]

[b] Techniques not listed in the Abraham & Michie (2008) taxonomy.

References:

1. Abraham C, Michie S. A taxonomy of behavior change techniques used in interventions. Health Psychol 2008;**27**(3):379-87.
