# Supplementary material for: Optimising self-care support for people with heart failure and their caregivers: development of the Rehabilitation Enablement in Chronic Heart Failure (REACH-HF) intervention using intervention mapping
Source: Pilot Feasibility Stud. 2016 Aug 2;2:37. doi: 10.1186/s40814-016-0075-x (PMC5153822; doi:10.1186/s40814-016-0075-x)
Supplement: Additional file 4: — The REACH-HF Facilitation Process. (PDF 280 kb) [file 40814_2016_75_MOESM4_ESM.pdf]

Additional File 4: REACH-HF Facilitation Stages

Stage 1 – Introducing the intervention

This stage is characterised by a meshing of the facilitator's (health) agenda with that of the patient (and caregiver)'s individual agenda to produce a joint approach regarding the nature and pace of the intervention.

- Needs assessment/appraisal of patient (and caregiver)'s current situation including some history taking.
- Eliciting patient's key priorities – using the manual topics to facilitate these if the patient does not identify issues.
- Manual is introduced – emphasis on reading the manual in bite-size / 'cup-of-tea size' chunks.
- Progress tracker is introduced – main emphasis on traffic lights, completing daily weight section, and exercise section. (NB. Other sections may also be highlighted and discussed in the first session.)
- Physical activity programme discussed and agreed. CBE DVD introduced as applicable. Initial exercise goal set.

(Other topics may be discussed and goals set depending on the individual needs and capacity of the patient.)

- (Where applicable) caregiver resource introduced and caregiver involvement emphasised.

Stage 2 – During the intervention period

This stage is characterised by the patient (and caregiver) learning how to engage with the facilitation role, how to use the materials, and individual application of new learning and behaviours.

- An initial treatment plan is identified and agreed. This is responsive to the individual.
- The facilitator continues to signpost to the manual as well as having in-depth discussions about topics.
- The facilitator encourages use of the materials – including Progress Tracker, DVD and CD (as applicable).

- The progress tracker is used for monitoring and review.
- New patient priorities may be identified and incorporated into the intervention plan (either addressed directly or via onward referral).
- Problem solving and revisiting key topics as required.
- Caregiver involvement and caregiver needs explored and responded to.

Stage 3 – Bringing the intervention to a close

This stage is characterised by review and reflection, and future and forward planning.

- Prior to the final session the facilitator reminds the patient (and caregiver) that the intervention is coming to an end.
- The facilitator encourages the patient (and caregiver) to identify any unaddressed issues prior to the final session.
- Reflection and review are used to highlight and celebrate progress made, and to identify skills and approaches that will help deal with any setbacks and with long-term maintenance.
- Future planning includes:
    - goal setting (to continue with current behaviours e.g. daily weighing, and/or inclusion of new ones e.g. increase exercise level – if applicable)
    - withdrawal of facilitator support and signposting to who to contact if further support is required (e.g. HFSN, GP, carer support group)
    - strategies for managing setbacks
    - self-monitoring strategies e.g. where to record information previously recorded in tracker
    - ongoing use of the manual as a reference guide
