# Supplementary material for: Optimising self-care support for people with heart failure and their caregivers: development of the Rehabilitation Enablement in Chronic Heart Failure (REACH-HF) intervention using intervention mapping
Source: Pilot Feasibility Stud. 2016 Aug 2;2:37. doi: 10.1186/s40814-016-0075-x (PMC5153822; doi:10.1186/s40814-016-0075-x)
Supplement: Additional file 5: — Rehabilitation Enablement in Chronic Heart Failure (REACH-HF) Feasibility Study Report. (PDF 563 kb) [file 40814_2016_75_MOESM5_ESM.pdf]

Additional File 5: Rehabilitation Enablement in Chronic Heart Failure (REACH-HF) – Feasibility study

A multicentre single-arm intervention study with parallel process evaluation to assess the feasibility and acceptability of the REACH HF manual for patients, facilitators and caregivers.

END OF STUDY REPORT [with outcome data at follow up redacted]

Final Version: 28th April 2015

Report compiled by Anna Sansom, Jenny Wingham, Colin Greaves and Rod Taylor

PenCTU Study Number:	2012/004
REC Reference:	13/SC/0640
ISRCTN number:	ISRCTN25032672

**Study Sponsor:**	Royal Cornwall Hospitals NHS Trust
**Chief Investigator:**	Professor Rod Taylor (University of Exeter Medical School)
**Co-Chief Investigator:**	Dr Hayes Dalal (Royal Cornwall Hospitals NHS Trust & University of Exeter Medical School)

CONTENTS PAGE

1. EXECUTIVE SUMMARY 3

2. INTRODUCTION 5

3. METHODS 7

4. RESULTS 14

4.1 Patient flow and baseline demographic data 14

4.2 Intervention Feasibility & Acceptability 17

4.3 Fidelity of manual delivery 18

4.4 Patient and caregiver outcome results 18

4.5 Patient/caregiver perception of trial processes 21

5. CONCLUSIONS 22

6. APPENDICES: 23

1. EXECUTIVE SUMMARY

Background

The REACH-HF feasibility study was a multicentre single-arm feasibility study with parallel process evaluation to assess the feasibility and acceptability of the REACH HF manual for systolic HF patients, their caregivers and facilitators delivering the intervention. The feasibility study was conducted in preparation for a fully powered randomised controlled trial assessing the clinical effectiveness and cost effectiveness of the HF Manual vs. usual care in patients with systolic HF and a separate single centre pilot trial in patients with HFpEF.

Study aims

Research aims:

- To assess the feasibility and acceptability of the addition of the HF Manual to usual care for systolic HF patients, caregivers and intervention facilitators.
- To assess the fidelity of HF Manual delivery by intervention facilitators.
- To evaluate components of the study process to inform the main randomised controlled trial design: feasibility of outcome data collection processes, and outcome burden and completion/attrition rates for patients and caregivers.

Intervention development aims:

- To identify any further training needs for the intervention facilitators.
- To identify any changes needed in the HF Manual.
- To finalise the content and format of the HF Manual and training materials

This report provides a summary of the outcome of the REACH feasibility study against the research aims.

Methods

The study used a four centre (Birmingham, Cornwall, South Glamorgan and York) single-arm design with a parallel process evaluation. Following identification and recruitment, patients with systolic HF received the HF manual intervention which was delivered over a period of 12 weeks by trained intervention facilitators, in addition to their usual care. The parallel process evaluation utilised qualitative methods and an observed structured clinical assessment using an intervention fidelity checklist in the context of delivering the REACH HF Manual to people with systolic HF. Multiple rounds of data collection and interaction with the intervention facilitators delivering the intervention generated feedback that informed both changes to the HF Manual, and changes to the training materials (i.e. the way the HF Manual is delivered by the intervention facilitators). The patient and caregiver outcome measures planned for the main trial of clinical effectiveness and cost-effectiveness were collected pre- and post- intervention in this study, in order to test procedures for collection, burden for patients, completeness of data collection and the rate of patient attrition/loss to follow up.

Results

Recruitment of patients and their caregivers took place over the 12 week period from 1st March 2014 to 31st May 2014, a one month extension on the planned 2-month period due to delayed excess treatment costs (ETC) agreement and delayed recruitment start at one centre (York). A total of 23 patients and 12 caregivers were recruited – meeting the recruitment target for the study. A total of 7 intervention facilitators were recruited.

- REACH-HF manuals appear to have been well accepted by patients, caregivers and facilitators.
- Patients and caregivers were highly satisfied with REACH-HF intervention.

- There was a need for some modifications to the manual content and format & facilitator training.
- Fidelity scoring indicated adequate delivery for most aspects of the intervention by all the facilitators
- Two items (addressing emotional consequences of being a caregiver and caregiver health and well-being) need reinforcement in future intervention delivery.
- There were generally excellent levels of outcome completion and patients/caregivers perceive relatively low outcome burden.
- A number of patient and caregiver outcomes following REACH-HF intervention showed evidence of improvement (with all caveats of a small population of selected participants and the study design of pre-post comparison with no control group).
- No safety issues were identified.
- The incremental shuttle walk test (ISWT) was not universally popular with patients and failed to show change over time

Conclusions
Following the feasibility study and discussion with the Programme Steering Committee (PSC), a small number of revisions to the main trial processes were implemented and are detailed in this report.

2. INTRODUCTION

Heart failure (HF) is becoming more prevalent worldwide and in the UK around 900,000 people have HF. Two editorials in the *Lancet* (2011) highlighted the current challenges of HF management, including the need for patients to be admitted to hospital or receive specialist care for extended periods, and the resulting financial costs of up to £1 billion per year.

There is no 'cure' for HF. Treatment includes medication and a range of self-management activities including exercise, planning and pacing activities of daily living, adjusting medication dosage, managing the emotional consequences of having heart failure, monitoring for signs and symptoms of deterioration, and communicating with health professionals.

Cardiac rehabilitation (CR) can be defined as 'the process by which patients with cardiac disease, in partnership with health professionals, are encouraged and supported to achieve and maintain optimal physical health'. A Cochrane systematic review of exercise-based CR for HF identified important quality of life benefits in participants, as well as reductions in HF admissions compared with usual care. Based on this evidence, in 2010 the National Institute of Health and Care Excellence (NICE) recommended offering CR based on supervised group exercise CR for people with both systolic and diastolic HF. Despite this recommendation, our recent survey indicates that few UK centres (16% of those surveyed) currently have a specific CR programme for those with HF. The UK uptake of CR for people with HF therefore remains poor. We believe an important potential solution to this poor provision and uptake is the development of a home-based self-help CR manual designed to meet the needs of people with heart failure and their caregivers.

REACH-HF is funded by a Programme Grant for Applied Research (reference number RP-PG-1210-12004) awarded by the National Institute for Health Research. Details of the various REACH HF work packages are available elsewhere (http://www.rcht.nhs.uk/RoyalCornwallHospitalsTrust/WorkingWithUs/TeachingAndResearch/ReachHF/ResearchProjects.aspx). The overarching aim of REACH-HF is to develop and evaluate a facilitated, home-based HF manual to enhance quality of life and self-management of people with HF and their caregivers.

Since the beginning of the REACH HF project in January 2013, the focus of the project has been the development of a facilitated HF manual according to MRC guidelines for complex interventions. The REACH-HF Manual has been developed using a systematic intervention development process called intervention mapping, theories of social support and behaviour change, and existing research evidence. The HF Manual development has been overseen by a Truro-based patient and public involvement (PPI) group that includes HF patients and caregivers. The core interventional components of the HF manual are exercise training/physical activity promotion, stress management, and education.

The REACH-HF feasibility study was a multicentre single-arm study with parallel process evaluation to assess the feasibility and acceptability of the REACH HF manual for systolic HF patients, their caregivers and facilitators delivering the intervention. The feasibility study was conducted in preparation for a fully powered randomised controlled trial assessing the clinical effectiveness and cost effectiveness of the HF Manual vs. usual care in patients with systolic HF and a separate single centre pilot trial in patients with HFpEF.

The feasibility study has both research aims and intervention aims, as detailed below;

Research aims:

- To assess the feasibility and acceptability of the addition of the HF Manual to usual care for systolic HF patients, caregivers and intervention facilitators.
- To assess the fidelity of HF Manual delivery by intervention facilitators.
- To evaluate components of the study process to inform the main randomised controlled trial design: feasibility of outcome data collection processes, and outcome burden and completion/attrition rates for patients and caregivers.

Intervention development aims:

- To identify any further training needs for the intervention facilitators.
- To identify any changes needed in the HF Manual.
- To finalise the content and format of the HF Manual and training materials

The purpose of this report is to provide a summary of the outcome of the REACH feasibility study against the research aims.

The impact of the feasibility study on intervention development aims is presented elsewhere: *'REACH-HF Feasibility Study Process Evaluation Interim Report – July 2014' and 'REACH-HF Feasibility Study Process Evaluation Final Report – September 2014'.* Reports compiled by Anna Sansom, Jenny Wingham and Colin Greaves.

3. METHODS

3.1 Study Design

The study used a four centre (Birmingham, Cornwall, South Glamorgan and York) single-arm design with a parallel process evaluation.

Following identification and recruitment, patients with systolic HF received the HF manual intervention which was delivered over a period of 12 weeks by trained intervention facilitators, in addition to their usual care. The parallel process evaluation utilised qualitative methods and an observed structured clinical assessment using an intervention fidelity checklist in the context of delivering the REACH HF Manual. Multiple rounds of data collection and interaction with the intervention facilitators delivering the intervention generated feedback that informed both changes to the HF Manual, and changes to the training materials (i.e. the way the HF Manual is delivered by the intervention facilitators). These changes are outlined in the separate feasibility study intervention development report[1]. The patient and caregiver outcome measures planned for the main trial of clinical effectiveness and cost effectiveness were collected pre- and post- intervention in this study, in order to test procedures for collection, burden for patients, completeness of data collection and the rate of patient attrition/loss to follow up.

3.2 Study populations

In this study, patients, caregivers and the intervention facilitators were considered participants. A sample size calculation was not performed as this study was examining feasibility objectives and was not powered to undertake inferential within-group analyses. The range of 16-24 patients and 4-8 intervention facilitators for this study was derived empirically based on the objectives of the study and to reflect what was practical across the four sites. Where possible, sites were requested to provide 2 intervention facilitators (8 in total) each with a case load of 3 patients (i.e. a total of 24 patients). It was considered that experience of providing several sessions for 3-4 patients each would give the facilitators sufficient exposure to the intervention to be able to comment on its feasibility and acceptability. Based on a previous trial of CR in HF patients and their caregivers we expected to include 11-17 caregivers in this study.

3.2.1 Patients: eligible patients were adults (aged ≥18 years), with a confirmed diagnosis of systolic HF on echocardiography (i.e. left ventricular ejection fraction < 45%) within the last 5 years, had been clinically stable for at least 2 weeks and in receipt of medical treatment for HF, were deemed suitable for exercise, and who did not have a contraindication to exercise (as adjudged by the PI in collaboration with the local clinical team with reference to the American Heart Association 2013 guidelines) and provided informed consent to take part.

Exclusion criteria were:

- Patients who had undertaken cardiac rehabilitation (CR) within the previous 12 months
- Patients who had received an intra-cardiac defibrillator (ICD), cardiac resynchronisation therapy (CRT), or combined CRT/ICD device implanted in the previous 6 months.
- Patients who were in a long term care establishment or who were unwilling or unable to travel to research assessments or accommodate home visits.
- Patients who were unable to understand the study information or unable to complete the outcome questionnaires.

[1] ‘REACH-HF Feasibility Study Process Evaluation Interim Report – July 2014’ and ‘REACH-HF Feasibility Study Process Evaluation Final Report – September 2014’. Reports compiled by Anna Sansom, Jenny Wingham and Colin Greaves.

- Patients who were judged to be unable to participate in the study for any other reason (e.g. psychiatric disorder, diagnosis of dementia, life threatening co-morbidity).
- Patients who were participating in concurrent interventional research which may have over-burdened the patient or confounded data collection.

3.2.2 Caregivers: patient's caregivers who were aged 18 years or older were invited to participate if they met the following definition: *'Someone who provides unpaid support to family or friends who could not manage without this help. This could be caring for a relative, partner or friend'* and provided informed consent to take part. Patients were still able to participate in the study if they didn't have an identified caregiver, or if the patient's caregiver was not willing to participate; the separate caregiver component of the HF manual was not applicable for such patients.

3.2.3 Intervention facilitators: the CR service in each of the four sites was asked to identify 1 or 2 members of the team to act in the capacity of the facilitator to deliver the HF Manual ('the intervention facilitator'). The intervention facilitator recruitment process specified that the intervention facilitator would be required to take part in the process evaluation. This included having consultations audio-recorded, taking part in a qualitative interview and completing questionnaires and providing other information as requested by the research team. Informed consent was obtained from the intervention facilitators.

3.3 Patient identification and recruitment processes

Patients were identified for this study using a three-pronged approach i.e. via primary care, secondary care and the local specialist HF nurse services.

Patients who expressed an interest in the study when returning the study reply form, were provided with an information pack which included a Study Invitation letter, a copy of the patient Participant Information Sheet (PIS) and a separate information pack for their caregiver (comprising a study invitation letter and a copy of the caregiver PIS). If the patient had an identified caregiver, they were prompted by their study invitation letter to pass the caregiver information pack to their caregiver for consideration. A member of the research team at the local site contacted the patient using the contact details provided by the patient on the reply form, once they had had at least 24 hours to read the study information. If the patient and their caregiver were still interested in participating, a screening assessment form was completed with them over the phone, enabling a provisional assessment of patient and caregiver eligibility. Provisionally eligible patients and their caregivers were then invited to the local investigator site for a baseline visit. Ineligible patients were provided with an explanation as to why they were not eligible to take part in the study at that time.

Patients were asked to attend two research clinics; one before (baseline) and one after the twelve week intervention period. A tabulated schedule of events for the study is provided in Appendix 1.

Following successful recruitment, consent was obtained from patients and caregivers at the baseline visit. After written informed consent was obtained by the PI (or authorised delegate), demographic and medical history information was collected from participating patients. Each participant was allocated a unique study number by which they were identified in all study-related documentation. Participating patients were then asked to complete a questionnaire booklet, perform an incremental shuttle walking test (administered by the PI or research nurse to assess exercise capacity), answer some questions relating to healthcare service utilisation over the prior three months, provide a blood sample for measurement of natriuretic peptide levels and wear an accelerometer for seven days. Patients were asked to return the device (via stamp addressed package) after seven days. Participating caregivers were also asked to complete a questionnaire booklet at the initial visit.

3.4 Intervention

The intervention was the REACH-HF facilitated heart failure manual in addition to usual care that includes treatments and management as defined by current NICE guidelines (2010) and local practice. The REACH-HF intervention is a bespoke novel evidence-based intervention developed by the REACH-HF team.

The HF manual comprises a self-help manual which patients worked through with facilitation by the REACH-HF intervention facilitator. The manual includes information and interactive elements relating to a wide range of topics relating to living with/adapting to living with heart failure, and covers four core elements:

(1) An exercise training programme based on a walking programme or a chair-based exercise DVD, or a combination of the two (the patient's choice);
(2) Stress management
(3) Medication management.
(4) Monitoring and managing symptoms (especially fluid build up)

Building a functional understanding of heart failure and the effects of self-care on symptoms and well-being is a further key target which applies to all the above goals.

In addition, patients were given a 'Progress Tracker' booklet and encouraged to record weekly activities to monitor and manage their symptoms to track The REACH-HF intervention facilitator worked to build the patient's and the caregiver's understanding of their situation and how to manage heart failure. The manual provides a wealth of information to facilitate increased knowledge and coping skills. The caregiver resource includes three chapters; providing support, becoming a caregiver and getting help.

The intervention facilitators were trained by the REACH-HF team in a three-day training course including both written and interactive (face-to-face) training materials. They also received monthly supervision by teleconference from members of the intervention development team. This provided opportunity to share feedback about intervention delivery and to provide updates and advice to refine intervention delivery procedures during the course of the study.

The REACH-HF facilitators were not responsible for adjusting the patient's medication. However, if during a consultation, the patient or caregiver reported side-effects that might be associated with the patient's current medical therapy; these were reported to the patient's GP or appropriate service such as an HF nursing service. Similarly, if during a visit the intervention facilitator considered a patient to be unwell, and that immediate medical care was required, the facilitator immediately made a referral to the appropriate health professional, or in the case of an emergency to the emergency services. These practices were specified in the training materials for the intervention facilitators.

Participating patients and their caregivers were contacted by a local intervention facilitator following completion of the screening/baseline visit in order to arrange the initial home visit. At the initial visit, patients and caregivers were provided with their copies of the HF Manual and were given a detailed introduction by the intervention facilitator. During the 12 weeks that followed, the intervention facilitator conducted a minimum of two further visits to the patient's home (which the caregiver was also asked to attend) and typically made 4 telephone contacts. The number of contacts, timing and contact time varied depending on the patient's needs.

3.5 Process evaluation

Audio recordings of all home-visit and telephone interactions between the intervention facilitator and patients/caregivers were made by the intervention facilitator. Patients and caregivers consented for these audio recordings to be made as part of their informed consent. As part of the process evaluation, patients and caregivers were also asked to complete questionnaires asking them to rate their satisfaction with the HF Manual. These questionnaires assessed feasibility, satisfaction and ideas for improvement and were given a) after each face-to-face session and b) at 3 months (end of

intervention/the last facilitator visit). These also included some questions about any telephone contacts that they received between visits. A similar questionnaire was given to the intervention facilitators as part of the ‘facilitator contact sheet’. The questionnaires contained mainly open-ended questions and a single satisfaction/overall feasibility question with a Likert response scale. The facilitator contact sheet was completed for each patient contact to record basic attendance and contact time. The contact sheet asked the intervention facilitator to make notes immediately after the patient contact about what was covered, what went well in the session; what worked less well; what s/he could have done differently and what could be improved about the intervention materials or the delivery process.

Process evaluation data collected during the 12 week intervention period was the primary data used to assess intervention fidelity. The procedure involved: a) identifying the change techniques and delivery processes that were associated with the intervention (as defined by prior intervention mapping) and b) using the Dreyfus skill acquisition scale to rate the competence of providers in delivering the targeted techniques and delivery processes. This produced a score of 0 to 6 for each targeted element of the intervention process. The scale was anchored such that a score of 3 was considered acceptable, 0 was non-existent and 6 was perfect performance. Two experts in behaviour change intervention listened to the set of recordings for each patient and applied the fidelity measure to rate competence in each element of intervention in each session and across the whole set of recordings. They marked sections of the recording that were useful for informing feedback to the facilitators (examples of good or poor practice, or which illustrated the intended intervention processes). This allowed the construction of formative feedback and prompts for discussion for each intervention provider. These sections were occasionally transcribed so that they could be used to inform future training. Where processes were consistently difficult to deliver and performance did not improve with feedback, or if the facilitators felt strongly that part of the intervention was not working/not workable, this suggested changes to the HF Manual or to the delivery procedures.

A semi-structured supervision session for the intervention facilitators was provided once every 4 weeks during the delivery period (3 in total). This was an opportunity to share ideas/good practice, problem-solve, and provide and discuss formative feedback based on the review of recorded sessions. The importance of completing the facilitator contact sheets was also reinforced during these sessions. The supervision sessions were conducted by teleconference to pool learning across the 4 sites and these sessions were also recorded. The last supervision session was run as a focus group targeted specifically at identifying ways to improve the intervention and the training. A topic guide was developed during the study, based on the feedback received in the earlier stages of data collection.

Brief semi-structured interviews, either by telephone or face-to-face, were conducted with 12-16 patients and their caregivers about their experiences of receiving the HF manual after 6 weeks (half way through intervention period) and between week 13 and 17 (the end of the intervention period). Topic guides were developed in consultation with the intervention development team, the REACH HF co-applicants, and the PPI group.

On completion of the 12-week intervention delivery period, participating patients and caregivers attended a second and final research clinic, overseen by the PI. As part of their assessment, participating patients and caregivers completed the assessments they completed at the initial baseline visit. In addition, patients were asked to identify contacts with healthcare and personal social services contacts during the intervention delivery period. For all hospitalisations and incidence of death, the hospital discharge letter and/or death certificate was obtained by the PI (or delegate), anonymised and sent to the Peninsula Clinical Trials Unit (‘CTU’). These records were subsequently

made available to an independent event adjudication committee and were used for safety reporting procedures.

Each patient and caregiver recruited was involved in the study for approximately 4 months between initial approach and clinic visit 2. A sample of patients participated for longer as they were selected for interview.

3.6 Outcome measures

The outcome measures planned for the definitive REACH HF trial in systolic HF {Work Package 3) and pilot REACH-HFpEF (Work Package 2) trials were collected from patients and caregivers in this feasibility study to allow researchers to test processes for collection and to assess the outcome completion rate. We also assessed outcome burden using the Trial Process Questionnaire which was completed by patients and caregivers (if appropriate) during the second research clinic visit.

The proposed primary outcome for the definitive and pilot trials is disease specific health related quality of life (HRQoL) measured using the Minnesota Living with Heart Failure Questionnaire (MLHFQ) in patients. Secondary outcome measures include: composite outcome of death or hospital admission related to HF or not related to HF (patients), blood natriuretic peptide levels (patients), Incremental Shuttle Walk Test (ISWT) (patients), Hospital Anxiety and Depression Scale (HADS) (patients & caregivers), physical activity level (obtained from accelerometer worn over two 7-day periods) (patients), EQ-5D (patients), HeartQoL (patients), SCHFI (caregivers), CBQ-HF (caregivers), CC-SCHFI (caregivers), FAMQOL (caregivers), healthcare utilisation (patients), safety outcomes (patients). All outcomes were assessed by clinic visit at baseline (visit 1) and at 3-months (visit 2). Outcome questionnaires were self-completed with the exception of healthcare utilisation and safety that were administered by the PI/research nurse.

This study assessed the feasibility and acceptability of collecting data on participant NHS and related resource use (self-report and/or routine/electronic records) to inform the health economic evaluation to be undertaken in the definitive and pilot trials. During the feasibility study we have developed and tested methods for estimating the resource use and costs associated with delivery of the intervention (e.g. via case report forms, and/or interviews with intervention providers). Levels of data completion on these outcomes were assessed.

All serious adverse events were recorded regardless of relatedness; non-serious, unrelated adverse events were not recorded. HF-related hospital admissions and HF-related death was a clinical outcome measure collected during this study. In order to collect this outcome measure data, the PI (or authorised delegate) obtained a death certificate or hospital discharge summary for every hospitalisation or death reported as a serious adverse event. Documents pertaining to a hospitalisation or death were anonymised prior to being sent to the CTU for subsequent adjudication by an independent event adjudication committee.

Safety information pertaining to caregivers was not be collected or reported since risks to caregivers were expected to be negligible. However, the protocol stipulated that any safety concerns identified through caregivers' completion of questionnaires or interviews were duly managed and reported.

A table summary of the outcome collection timings is detailed in Appendix 1.

3.7 Data management

Data was recorded on study specific data case report forms (CRFs), usually by the research team at each site. All persons authorised to collect and record trial data at each site were listed on the study site delegation logs, signed by the relevant PI. Source data included all data recorded straight into the CRF, blood test result forms, accelerometer data, death certificates (if applicable), discharge

summaries (if applicable), and any patient completed questionnaire booklets. Completed CRFs were transferred to the CTU for double-data entry onto a password-protected database. All forms and data were tracked using a web-based trial management system. Double-entered data was compared for discrepancies using a stored procedure. Discrepant data was verified using the original paper data sheets.

3.8 Data analysis

3.8.1 Qualitative analysis

Audio files and transcriptions of the data were collected by the Process Evaluation Team, comprising REACH-HF team co-applicants and collaborators.

The analysis of audio recordings (of sessions and interviews, supervision sessions and patient interviews) did not rely on the recordings being transcribed. The audio files were listened to and notes taken by the researcher and also by one of the intervention development team. The purpose of analysing this data was not to produce a robust, in-depth qualitative analysis, but to provide sufficient feedback on the intervention to inform refinements to the intervention and training materials. Analysis of raw audio recordings, rather than transcribed data was considered sufficient for this purpose. The audio files were stored on secure servers at the University of Exeter and the Royal Cornwall Hospitals Trust. Access was password protected and limited to the REACH-HF study team.

Intervention fidelity checklist scores were summarised using simple descriptive statistics (means and standard deviations) and collated both in total and by facilitator. Examples of good practice were flagged, transcribed and extracted as audio clips (with the facilitator's permission) to inform future training. Checklist items were scored for each recording and as a summary score (in relation to the full set of available recordings for each patient). Low-scoring items for some or all facilitators identified areas where change was needed (either via formative feedback and a training update or via changes to the intervention materials). The checklist scores and notes taken about good practice and learning needs during the review of the recordings provided ideas for individualised formative feedback to each facilitator (in written form). The methodology had been used successfully before in previous complex intervention trials undertaken by the applicants.

Feasibility and acceptability was assessed via: a) data from patient and caregiver interviews and from recordings of the supervisory meetings and b) patient and facilitator feedback questionnaires, which included satisfaction scores as well as open-ended feedback on what worked well and what improvements could be made. The qualitative data was analysed using descriptive, thematic analysis to identify salient themes from the interview transcripts and open-ended sections of the questionnaires. The data was analysed with the aim of addressing the research aims above, in particular to see if patients, caregivers and facilitators have different views, and to extract recommendations for changes needed to the intervention manual and/or the intervention delivery processes.

3.8.2. Quantitative analysis

Given the feasibility nature of this trial we did not propose to formally inferentially test differences in outcomes and costs within groups. Mean and standard deviation for primary and secondary outcomes were reported at baseline and 3 months' follow up. Participant flow through the study was summarised using the CONSORT diagram (adapted for complex interventions) and reflects the number of recruitment letters sent, numbers consenting, numbers participating , number undertaking intervention, and number of completed outcomes. Attrition rates were calculated with 95% confidence intervals

Figure 1 Overview of study design

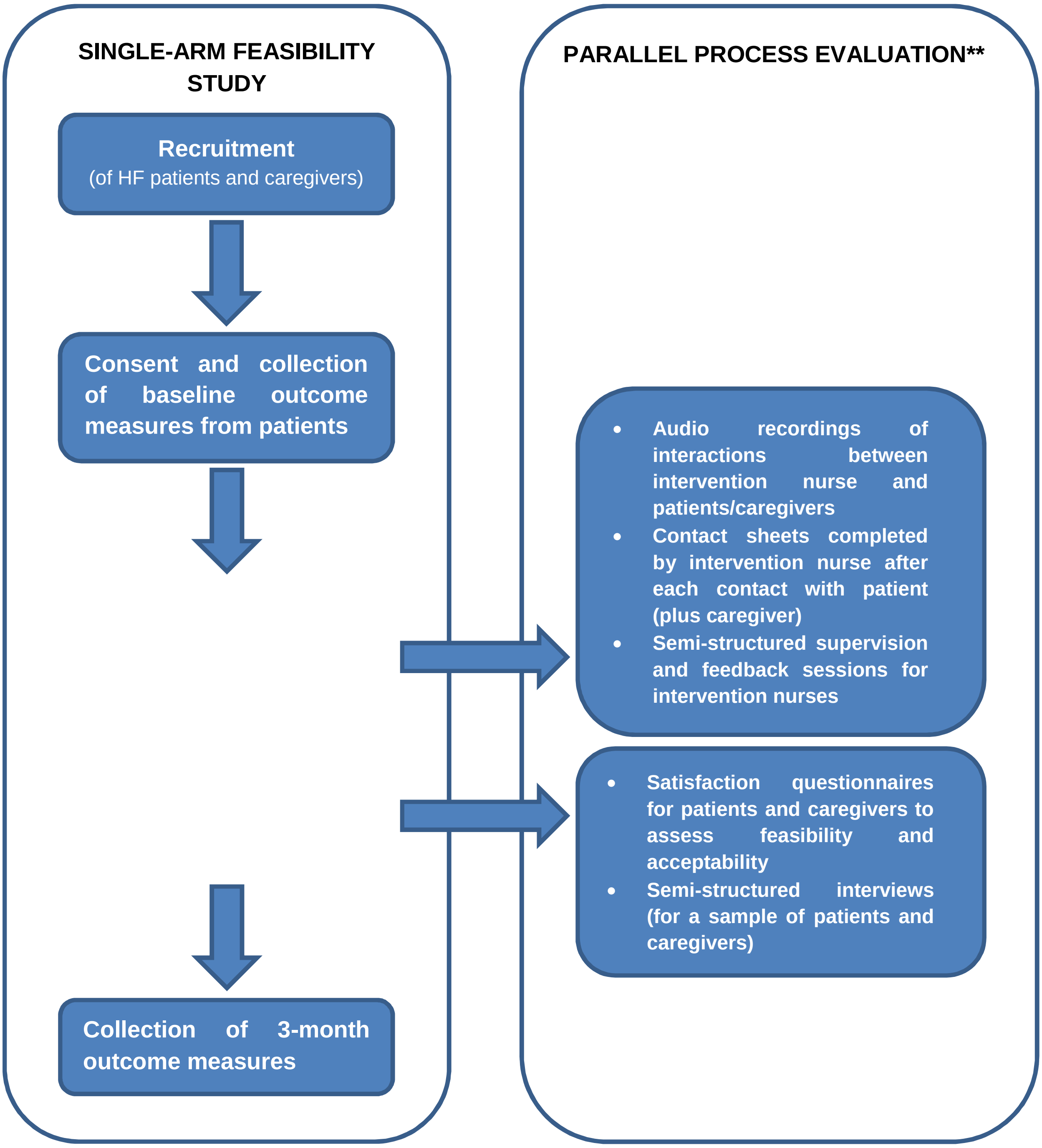

4. RESULTS

4.1 Patient flow and baseline demographic data

Study enrolment, allocation to intervention and follow-up of study patients and caregivers is summarised in Figure 1.

4.1.1 Patients and caregivers approached

Recruitment of patients and their caregivers took place over the 12 week period from 1st March 2014 to 31st May 2014, a one month extension on the planned 2-month period due to delayed ETC agreement and delayed recruitment start in York. The numbers approached and recruited are summarised in Table 1 and were fairly consistent across sites. Of the 84 patients approached, 23 were recruited i.e. an approach to recruit ratio of ~4:1.

4

4.1.2 Rates of recruitment

Following approaches to 84 patients, a total 23 patients and 12 caregivers were recruited – meeting the recruitment target for the study. For the period of time that sites were open, the overall study recruitment rate across the 4 sites was 10 patients/month and 5 caregivers/month. Rates of patient recruitment across the four sites are summarised in Table 1. The target recruitment rate for the study was 4-6 patients per month per site.

Table 1. Patient recruitment overall and across sites

	Total	B/ham	Gwent	Truro	York
Patients approached	N=84	N=17	N=16	N=45	N=6
Patients recruited<br>Monthly rate	N=23 (46%)<br>**10.0**	N=5 (29%)<br>**2.7**	N=7 (44%)<br>**4.3**	N=7 (16%)<br>**3.4**	N=4 (67%)<br>**5.3***
Carers recruited<br>Monthly rate	N=12<br>5.2	N=2<br>1.1	N=6<br>3.7	N=2<br>1.0	N=2<br>2.6

*recruitment was only open in York for 23 days

Figure 2 shows the target versus actual recruitment for patients. A total of 7 intervention facilitators were recruited slightly higher that the planned 2 per site.

Figure 1. Study flow

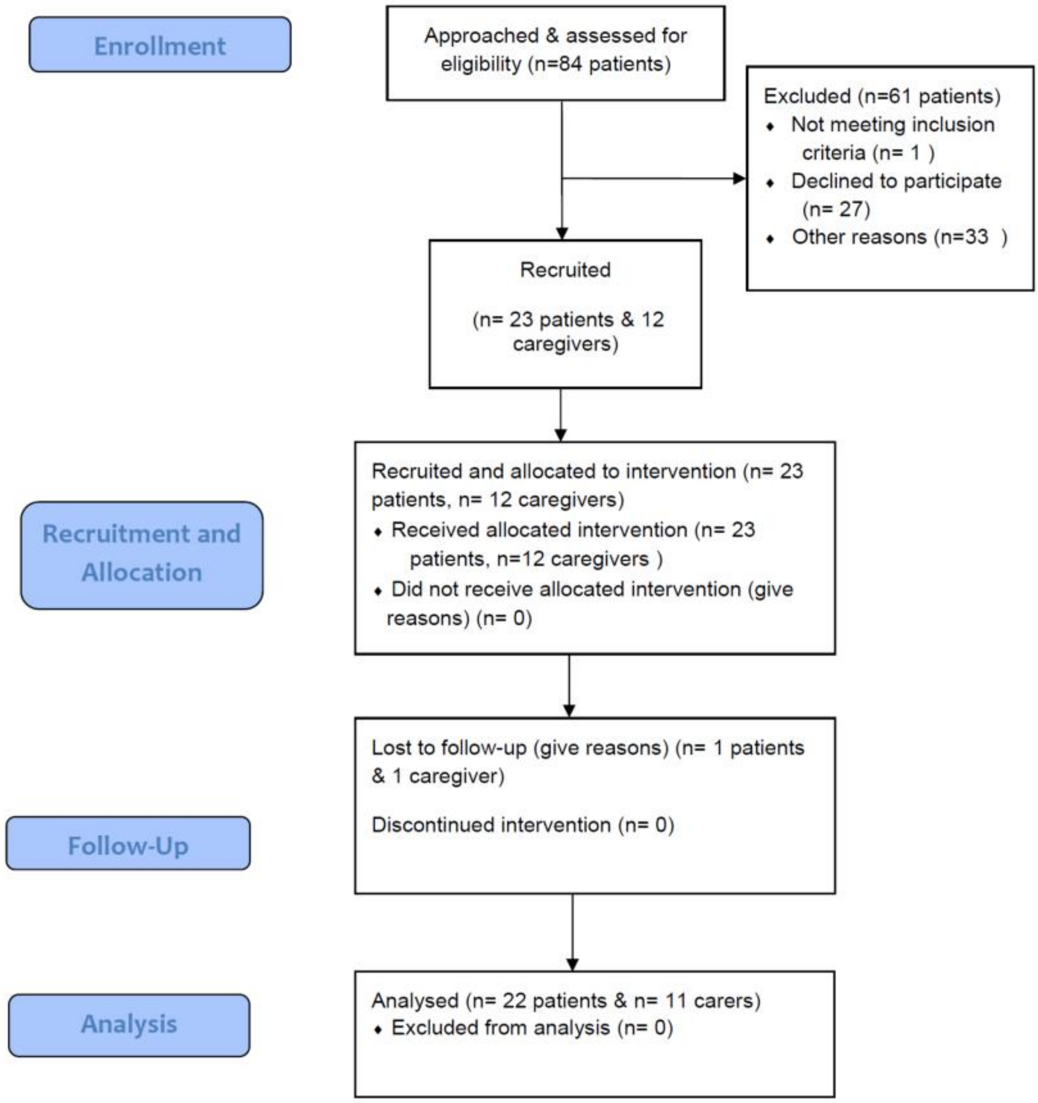

Figure 2. Study recruitment – target vs actual

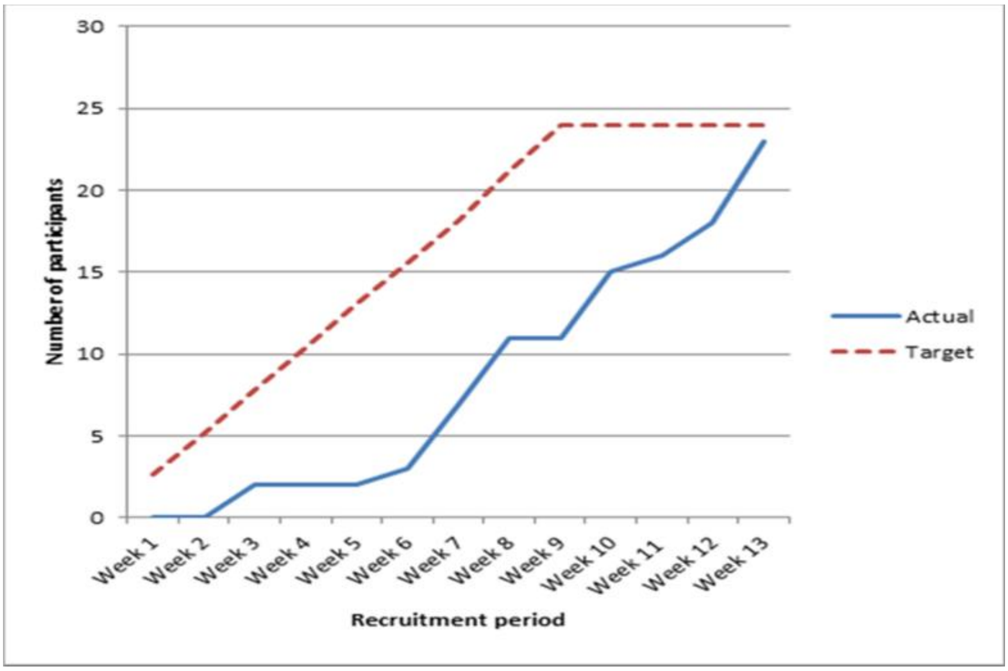

4.1.3 Baseline demographics of recruited patients

The baseline characteristics of the included 23 patients and 12 caregivers are summarised in the tables below.

Table 2. Baseline demographics of patients (N=23)

**Characteristic**	**N (percent)<br>or mean (SD) [range]**
Male	16 (70)
Age (years)	66 (14) [38 to 83]
BMI	32.2 (6.9) [23.1 to 53.0]
Smoking status<br>Current smoker<br>Ex-smoker<br>Never smoked	 0 (0)<br>13 (57)<br>10 (43)
NYHA status<br>Class I<br>Class II<br>Class III<br>Class IV	 2 (9)<br>15 (65)<br>6 (26)<br>0
Baseline use of medication (N=19)<br>Beta-blocker<br>Alpha 2 antagonist<br>ACE inhibitor	 18 (95)<br>6 (32)<br>13 (68)
Diagnosis of HF (years)<br>< 1<br>…1-2<br>…>2<br>…not available	 11 (48)<br>4 (17)<br>3 (13)<br>5 (22)
Main activity<br>In employment or self-employment<br>Retired<br>Housework<br>Other+	 5 (22)<br>16 (70)<br>1 (4)<br>1 (4)
Undertaken post school education	15 (65)

Table 3. Baseline demographics – caregivers (N=12)

**Characteristic**	**N (percent) or mean (SD)**
Male	4 (33)
Age	63 (14) [36 to 84]
Relationship to patient<br>Partner	 12 (100)

4.2 Intervention Feasibility & Acceptability

The quantitative and qualitative data collected to assess intervention feasibility and acceptability can be summarised as follows:

- Satisfaction questionnaire
  - N = 50 patient/caregiver questionnaire replies: mean score = 1.9 (see Table 3 below)
  - First questionnaire (first 1 to 2 weeks): mean 2.3 (median 2) & last questionnaire (last 10-12 weeks): mean 1.7 (median 2)

Table 3. Summary of patient/caregiver satisfaction scores (first 50 questionnaires)

1 Excellent	16
2 Very good	26
3 Good	4
4 Satisfactory	3
5 Poor	1
6 Very poor	0

- Facilitator contact sheets: N = 18 patients completed interventions
- Mean number of sessions = 8 (median 8, range 6 to 11)
- Mean duration = 346 minutes (median 338, range 110 to 583)
- Patient tracker: all patients (15/15) used exercise record section (but degree of completion very variable and lesser proportion completion for other sections)
- Intervention drop out: Nil
- Interviews: facilitators, patients & caregivers overwhelmingly positive (albeit specific recommendations for adaption of manual content, presentation & training). The following is a sample of patient quotes:

“Thank you for inviting me to take part. I feel so much more confident about managing my condition and I intend to keep active and keep improving my level of fitness. Thank you again.”
“(The facilitator) has gone over things in a way my partner and I find brilliant. A really brilliant explanation of anything I have asked.”
“(The facilitator) has been a great help and I am beginning to feel much better as time goes on.”
“I have found everything brill and the support from (the facilitator) excellent. This has already made a difference.”
“Having (the facilitator) visit gives me confidence… I feel more at ease about myself now”.
“The facilitator has provided us with the knowledge that we can be positive about the future”.
“We both feel more positive now about what we are dealing with and how to enjoy certain experiences”

4.3 Fidelity of manual delivery

Intervention fidelity was checked by applying our 13-item intervention fidelity checklist to all recorded intervention sessions (i.e. all the phone and face-to-face sessions for the 18 participants for whom data was returned). Table 4 shows the mean intervention fidelity scores and range of scores for each item and Table 5 shows the scores for each facilitator. The scores indicated adequate delivery (defined as a score of 3 or more) for most aspects by all facilitators. However, the mean score for items 10 (*addressing emotional consequences of being a caregiver*) and 11 (*caregiver health and well-being*) was less than 3. Analysis of the scores for each facilitator show that only one of the six facilitators delivered these elements of the intervention as intended.

Table 4: Mean intervention fidelity scores

	**Item 1**	**Item 2**	**Item 3**	**Item 4**	**Item 5a**	**Item 5b**	**Item 6**	**Item 7**	**Item 8**	**Item 9**	**Item 10**	**Item 11**	**Item 12**
N	18	18	18	18	18	18	18	18	18	15	15	15	18
Minimum	3.0	3.5	3.0	2.0	3.0	3.0	3.0	2.5	2.0	2.0	.0	.0	.0
Maximum	6.0	5.5	5.0	6.0	6.0	6.0	6.0	5.5	6.0	5.0	4.5	5.0	6.0
Mean	5.056	4.611	4.361	4.250	4.639	4.667	4.611	4.472	4.194	3.800	2.700	2.567	3.583
SD	.6157	.6543	.7237	.8952	.7031	.6642	.7962	.8309	1.1775	1.0657	1.4736	1.635	1.458

Table 4: Mean intervention fidelity scores by facilitator

		IF Score Item 1	IF Score Item 2	IF Score Item 3	IF Score Item 4	IF Score Item 5a	IF Score Item 5b	IF Score Item 6	IF Score Item 7	IF Score Item 8	IF Score Item 9	IF Score Item 10	IF Score Item 11	IF Score Item 12
		Mean	Mean	Mean	Mean	Mean	Mean	Mean	Mean	Mean	Mean	Mean	Mean	Mean
Facilitator ID	1	5.1	3.9	3.5	3.1	3.9	4.0	3.9	3.4	2.5	3.5	2.2	1.3	2.4
	2	5.3	4.2	4.0	4.2	4.5	5.0	4.7	4.5	3.8	3.8	2.5	2.3	3.3
	3	5.5	5.2	5.0	5.3	5.5	5.3	5.3	5.0	5.3	3.5	1.5	2.0	5.0
	4	5.0	5.1	4.8	4.5	4.8	4.5	4.4	5.0	4.5	5.0	4.1	4.4	4.0
	5	5.0	5.0	5.0	4.0	5.0	5.0	6.0	5.0	6.0	.	.	.	6.0
	6	4.3	4.7	4.5	4.5	4.7	4.7	4.7	4.5	4.7	2.7	2.3	2.0	2.7
	7	.	.	.	.	.	.	.	.	.	.	.	.	.

4.4 Patient and caregiver outcome results

[follow up data and text redacted]

4.4.1 Patient outcomes

The baseline (pre-intervention) and 3-month follow up results for the included patients are summarised in Table 5. With all caveats of this feasibility study (i.e. small population of selected participants and the study design of pre-post comparison with no control group) a number of patient outcomes following the REACH-HF intervention showed some evidence of improvement following intervention. The one exception was the lack of change in ISWT distance over time. The reasons for non-completion of the ISWT are provided in Appendix 2.

Adverse events are summarised in Table 6. Two serious adverse events requiring hospitalisation were seen during the 3 months of follow-up. One of these events was judged by the independent adjudication panel as HF-related and the other as non HF-related.

Table 5. Patient outcomes and baseline and 3-months

Outcome	Baseline<br>Frequency* (percent) or N, mean (SD) [range]	3-months follow up<br>Frequency* (percent) or N, mean (SD) [range]
Primary outcome		
MLwHF[I]		
Total score	22, 39.5 (24.6) [64 to 91]	[redacted]
Physical score	22, 10.1 (8.2) [0 to 25]	[redacted]
Emotional score	22, 19.0 (10.8) [4 to 40]	[redacted]
Secondary outcomes		
ISWT distance (m)[II]		
Practice	22, 265 (201) [40 to 780]	
Effort scale	*22, 4.9 (2.5) [0 to 10]*	
Main	16, 325 (226) [40 to 900]	[redacted]
…*Effort scale*	*15, 5.3 (2.2) [1 to 10]*	[redacted]
EQ-5D[II]		
Tariff	23, 0.61 (0.27) [-0.06 to 1.00]	[redacted]
Thermometer	21, 60.3 (17.8) [20 to 85]	[redacted]
Accelerometry[II]		
Ave mins/day light activity	17, 112 (64) [5 to 231]	[redacted]
....Ave mins/day at least light activity	17, 133 (77) [7 to 260]	[redacted]
…Ave mins/day at least moderate activity	17, 21 (23) [1 to 96]	[redacted]
….Ave mins/day vigorous activity	17, 0.1 (0.1) [0 to 0.4]	[redacted]
HADS[I]		
Depression score	23, 5.6 (3.3) [1.0 to 14.0]	[redacted]
Anxiety score	23, 7.3 (4.4) [1.0 to 18.0]	[redacted]
HeartQoL[II]		
Global score	23, 1.45 (0.78) [0 to 2.79]	[redacted]
Physical score	23, 1.28 (0.85) [0 to 2.70]	[redacted]
Emotional score	23, 1.86 (0.95) [0 to 1.93]	[redacted]
Self-care of Heart Failure Index (SCHFI)[II]		
Maintenance	23, 56.0 (13.5) [26.7 to 83.3]	[redacted]
Management **	13, 46.5 (20.4) [15.0 to 95.0]	[redacted]
Confidence	23, 58.2 (22.8) [11.1 to 100.0]	[redacted]
Deaths	-	[redacted]
Total hospitalisations	-	[redacted]
HF-related		[redacted]
Not HF-related		[redacted]
BNP level (pg/mL)[I]	15, 670 (468) [72 to 1439]	[redacted]

[I] Outcome where a lower score, indicates better outcome; [II] Outcome where a lower score, indicates better outcome ** There is no management total if there are any missing individual scores.

Table 6. Adverse outcomes in patients over 3-months follow up

Non-serious adverse events:

Description	Outcome	Severity	Relationship to research procedures / intervention
Nocturnal breathlessness/fluid overload	Resolved	Moderate	Unlikely
Chest pain	Resolved	Moderate	Possible
Urine infection	Resolved	Moderate	Not related

Serious adverse events:

Event	Brief summary	Outcome	Severity	Relationship to research procedures / intervention	Adjudication outcome	Further information
Hospitalisation	Troponin negative chest pain plus acute kidney injury	Recovered	Severe	Not related	Admission not due to heart failure	N/A
Hospitalisation	Planned admission due to deterioration of (heart failure) symptoms	Recovered with sequelae	Severe	Unlikely	Admission due to heart failure	Further investigation planned, CT thorax, follow up by HF Nurse & Cardiologist

Table 7. Caregiver outcome results at baseline and 3-month follow up

**Outcome**	**Baseline**<br>**Frequency* (percent) or**<br>**N mean (SD) [range]**	**3-months follow up**<br>**Frequency* (percent) or**<br>**N mean (SD) [range]**
HADS[I]		
Depression score	12, 5.2 (4.5) [1.0 to 17.0]	[redacted]
Anxiety score	12, 9.6 (6.7) [1.0 to 21.0]	[redacted]
Caregiver Contribution to Self-care of Heart Failure Index (CC-SCHFI)[II]		
Maintenance	12, 34.9 (22.4) [0.0 to 73.3]	[redacted]
Management **	8, 33.1 (11.3) [20.0 to 55.0]	[redacted]
Confidence	12, 48.1 (18.5) [16.7 to 77.8]	[redacted]
Caregiver Burden Questionnaire – Heart Failure (CBQ-HF)[I]		
Physical	12, 5.3 (5.7) [0.0 to 20.0]	[redacted]
Emotional	12, 22.6 (15.6) [4.0 to 52.0]	[redacted]
Social Life	12, 1.6 (2.3) [0.0 to 8.0]	[redacted]
Lifestyle	12, 5.2 (4.2) [0.0 to 15.0]	[redacted]
Family Caregiver-Specific Quality of Life Scale (FAMQOL)[I]		
Physical	12, 15.1 (3.2) [9.0 to 18.0]	[redacted]
Psychological	12, 12.2 (4.9) [5.0 to 20.0]	[redacted]
Social	12, 15.3 (3.3) [7.0 to 20.0]	[redacted]
Total	12, 56.3 (12.5) [29.0 to 74.0]	[redacted]

[I] Outcome were a lower score, indicates better outcome; [II] Outcome were a lower score, indicates better outcome ** There is no management total if there are any missing individual scores.

4.5 Patient/caregiver perception of trial processes

The table below present the questionnaire results from a questionnaire regarding trial processed completed patients and caregiver participants at end of the study. These data show that overall, participants found their involvement in the feasibility study to be a very positive one and there was no evidence of outcome completion burden.

Table 8. Summary of perception of trial process questionnaire

**Right amount of info collected at clinic?**	Too much	About right	Too little	
Patients (n = 19)	0	19	0	
Carers (n = 10)	0	10	0	
**Questionnaire completion problems?**	Yes	No		
Patients (n = 19)	1	18		
Carers (n = 9)	0	9		
**Overall impression of participation?**	Very Good	Good	Acceptable	Poor / Very Poor
Patients (n = 19)	14	5	0	0
Carers (n = 10)	9	0	1	0
**Research team helpful?**	Very helpful	Helpful	Okay	Unhelpful / Very Unhelpful
Patients (n = 19)	17	2	0	0
Carers (n = 10)	9	1	0	0
**Recommend participation to others?**	Strongly recommend	Recommend	Not recommend	Strongly not recommend
Patients (n = 19)	12	6	1	0
Carers (n = 10)	8	2	0	0

5. CONCLUSIONS

The conclusions of this feasibility study in terms of its research aims are summarised as follows:

Feasibility & acceptability of intervention

- REACH-HF manuals appear to have been well accepted to patients, caregivers and facilitators.
- Patients and caregivers were highly satisfied with REACH-HF intervention.
- There was a need for some modifications to manual content and format & facilitator training (see Appendix 3).

Fidelity of intervention delivery

- Fidelity scoring indicated adequate delivery for most aspects by all facilitators.
- Two items (addressing emotional consequences of being a caregiver and caregiver health and well-being) need reinforcement in future intervention delivery.

Trial processes

- Generally excellent levels of outcome completion and patients/caregivers perceive relatively low outcome burden.
- a number of patient and caregiver outcomes following REACH-HF intervention showed evidence of improvement (with the all caveats of a small population of selected participants and the study design of pre-post comparison with no control group).
- No safety issues identified.
- ISWT was not universally popular with patients and failed to show change over time

Following the feasibility study and discussion with the Programme Steering Committee, it was agreed that the following revisions to the trial processes be implemented:

- Reinforce/supplement outcome assessor training on the conduct of ISWT
- Review recruitment processes and plans with sites (i.e. patient information, recruitment monitoring) and identify 'backup' recruitment strategy(ies) in the event that recruitment is slower than expected
- Ensure that recruitment reflects the population of HF patients (in terms of age, disease severity)
- Extend baseline assessment to capture the full range of clinical descriptors
- Addition of EQ-5D for caregivers
- Modification to patient tracker (compliance measure of intervention compliance & analysis algorithm)
- Check accelerometry procedures (charging & transport) to minimise loss of data

Appendix 1: Outcome collection schedule

Study Schedule			
	Clinic visit 1 (Baseline*)	12 week treatment period	Clinic visit 2 (3 months)
Demographics (e.g. age, sex, NYHA class)	X		
Concomitant medication	X		X
Medical history	X		
Informed Consent	X		
Intervention delivery** (HF Manual)		X	
Process evaluation **		X	
MLHFQW	X		X
Hospital Anxiety and Depression Scale (HADS)	X		X
Self-care of Heart Failure Index (SCHFI)	X		X
Caregiver Burden Questionnaire – Heart Failure (CBQ-HF)	X		X
Caregiver Contribution to Self-care of Heart Failure Index (CC-SCHFI)	X		X
Heart-QOL	X		X
FAMQOL	X		X
Blood sample for natriuretic peptide levels	X		X
Shuttle walk test	X		X
Physical activity level (wear accelerometers for 7 days)	X		X
EQ_5D	X		X
Trial Process Questionnaire			X
Assessment of healthcare utilisation	X		X
Adverse events	X		X

Appendix 2. Reasons for non-completion of ISWT

0M ISWT1 - Not done Reasons

Reason	Freq
Diastolic >100, Systolic >180	1

0M ISWT2 - Not done Reasons

Reason	Freq
Shuttle walk 2 was not completed due to patient becoming wobbly and almost lost balance	1
Second walk test not performed due to patient experienced chest tightness	1
Second walk test not performed due to patient complaining of chest tightness	1
Patient found ISWT 1 difficult and was not keen to do ISWT 2	1
Patient felt it would be too much	1
BP 185/105	1
Systolic and diastolic BP greater than safe limits - not done	1

3M ISWT1 - Not done Reasons

Reason	Freq
Recently had surgery and unable to exercise for 6 weeks	1
DNA Visit	1

Appendix 3. Modifications to REACH HF manuals and training materials following feasibility study

Manual and materials

The Heart Failure Manual

- Include more testimonials particularly around relaxation/managing stress and managing changes in symptoms/ups and downs.
- Additional advice for people who are returning to work after a period of long term sick leave.

Progress Tracker

<u>NOTE:</u> We do not have a complete data set of end of intervention PTs (n=15/23), in addition, different facilitators may have placed differing emphasis on completing the PTs (some requesting that the patient did it 'to help the research' and others focusing on the benefit and appropriateness for the individual). Therefore it is suggested that the following recommendations be interpreted within this context.

- Ensure all sections have space for a full 12 week record.
- Review whether to include cause and specific advice in the 'My health care' section.
- Consider renaming 'Is it time to have some fun?' to e.g. 'leisure and fun'.

Other issues

- Give an indication of the timeframe for taking part in the research at the outset (including when to expect the first facilitator visit).
- Some sections had a negative tone: end of life and living with uncertainty sections – it was suggested that it could be a separate section for people it is more relevant for. Difficulty in feeling hopeful and positive from majority of HFM and then being 'brought down' by that section.

Training

Facilitator role

- Check time availability, preference, expectations and other commitments with participants before beginning the intervention. E.g. Is it realistic to have sessions that last for more than hour?

Progress Tracker

- If cause and specific advice in the 'My health care' section is to remain, reinforce in facilitator training re encouraging patients to complete this section (i.e. facilitators help patients to understand the benefit of using it).
- Emphasise in facilitator training the need to complete contact section on the Traffic Lights page.
- Emphasise that not all sections need to be completed: it is up to each individual patient to identify the most relevant and helpful sections for them. However, we may want to emphasise use of the weight, weekly progress, and exercise records (as a minimum) to focus on – in keeping with the aims of the intervention.

Other issues

- Where a facilitator had another role as HFSN there was potential for some ambiguity at the end of the intervention regarding whether the patient could still contact them or not. This led to some differences in how the facilitator ended the intervention and whether the patient +/or caregiver still felt supported by the same person. This distinction may be worth exploring more/being made more explicit.
- One participant's lifestyle did not allow them to complete the requests in the manual as s/he was also a caregiver for partner and friends.
